# Spatiotemporal depletion of tumor-associated immune checkpoint PD-L1 with near-infrared photoimmunotherapy promotes antitumor immunity

**DOI:** 10.1136/jitc-2021-003036

**Published:** 2021-10-28

**Authors:** Shunichi Taki, Kohei Matsuoka, Yuko Nishinaga, Kazuomi Takahashi, Hirotoshi Yasui, Chiaki Koike, Misae Shimizu, Mitsuo Sato, Kazuhide Sato

**Affiliations:** 1 Department of Respiratory Medicine, Nagoya University Graduate School of Medicine, Nagoya, AICHI, Japan; 2 Department of Integrated Health Sciences, Nagoya University Graduate School of Medicine, Nagoya, AICHI, Japan; 3 Advanced Analytical and Diagnostic Imaging Center (AADIC) / Medical Engineering Unit (MEU), B3 Unit, Nagoya University Institute for Advanced Research, Nagoya, AICHI, Japan; 4 FOREST-Souhatsu, CREST, JST, Tokyo, Japan; 5 Nagoya University Institute for Advanced Research, S-YLC, Nagoya University, Nagoya, AICHI, Japan

**Keywords:** antibodies, neoplasm, B7-H1 antigen, immunotherapy, tumor microenvironment, therapies, investigational

## Abstract

**Background:**

Near-infrared photoimmunotherapy (NIR-PIT) is a new modality for treating cancer, which uses antibody-photoabsorber (IRDye700DX) conjugates that specifically bind to target tumor cells. This conjugate is then photoactivated by NIR light, inducing rapid necrotic cell death. NIR-PIT needs a highly expressed targeting antigen on the cells because of its reliance on antibodies. However, using antibodies limits this useful technology to only those patients whose tumors express high levels of a specific antigen. Thus, to propose an alternative strategy, we modified this phototechnology to augment the anticancer immune system by targeting the almost low-expressed immune checkpoint molecules on tumor cells.

**Methods:**

We used programmed death-ligand 1 (PD-L1), an immune checkpoint molecule, as the target for NIR-PIT. Although the expression of PD-L1 on tumor cells is usually low, PD-L1 is almost expressed on tumor cells. Intratumoral depletion with PD-L1-targeted NIR-PIT was tested in mouse syngeneic tumor models.

**Results:**

Although PD-L1-targeted NIR-PIT showed limited effect on tumor cells in vitro, the therapy induced sufficient antitumor effects in vivo, which were thought to be mediated by the ‘photoimmuno’ effect and antitumor immunity augmentation. Moreover, PD-L1-targeted NIR-PIT induced antitumor effect on non-NIR light-irradiated tumors.

**Conclusions:**

Local PD-L1-targeted NIR-PIT enhanced the antitumor immune reaction through a direct photonecrotic effect, thereby providing an alternative approach to targeted cancer immunotherapy and expanding the scope of cancer therapeutics.

## Background

Near-infrared photoimmunotherapy (NIR-PIT) is a recently developed cancer treatment that uses antibody-photoabsorber conjugates and NIR light.^1 2^ Once the conjugates bind to the cell membrane, NIR light exposure selectively induces rapid cell-specific necrosis. A global phase III clinical trial of NIR-PIT is currently underway for treating inoperable recurrent head and neck cancers, which are targeted based on their overexpressed epidermal growth factor receptor (EGFR) (https://clinicaltrials.gov/ct2/show/NCT03769506). In September 2020, cetuximab-IR700 (ASP1929), an IR700-conjugated EGFR monoclonal antibody (mAb), was conditionally approved and registered for clinical use by the Pharmaceuticals and Medical Devices Agency in Japan.

NIR-PIT is a promising modality for selective cancer therapy; thus, a various tumor-cell surface protein-specific mAbs have been preclinically evaluated.^3 4^ As NIR-PIT relies on antibodies, it needs a highly expressed targeting antigen on tumor cells. However, using antibodies limits the application of this useful technology to only those patients who have highly expressed targeting antigens. Therefore, it would be highly desirable to modify NIR-PIT such that it kills tumor cells and simultaneously augments anticancer immunity.

The immune checkpoint protein programmed death-1 (PD-1) and its ligand PD-L1 are detected in various solid cancers; PD-1/PD-L1 blockade therapies have greatly improved clinical outcomes in various organ cancers.^5 6^ MAbs that block or bind to PD-L1 have been approved and are now widely used clinically.^7^ PD-L1 is found on tumor cell membranes. It dampens the effector T cell immune response on ligation, allowing immune surveillance evasion.^8^

Recently, it has been revealed that various inhibitory immune cells suppress T cell activation, such as regulatory T cells (Tregs), cancer-associated fibroblasts, alternatively activated macrophages, and myeloid-derived suppressor cells (MDSCs).^9–13^ MDSCs accumulate in the tumor bed, downregulating T cell activity and promoting tumor cell immune evasion.^14^ Therefore, modifying immune responses to reduce MDSC numbers in the tumor microenvironment could be a promising cancer immunotherapy strategy.^15 16^ MDSCs found in the tumor bed are reportedly associated with the PD-1/PD-L1 signaling axis and highly express PD-L1, whereas MDSCs in the lymphoid organs lowly express PD-L1.^17 18^ Thus, targeting PD-L1 may also affect MDSCs in the tumor microenvironment.

Here, we evaluated the antitumor effect of photoablation-mediated spatiotemporal PD-L1 depletion in a syngeneic mouse tumor model to realize a new NIR-PIT methodology targeting lowly expressed tumor proteins.

## Methods

### Study design

Our primary objective was to establish a new cancer immunotherapeutic strategy, which targeted tumor cells and modulated the antitumor immune system. Here, we demonstrated PD-L1-targeted cancer therapy, using a series of controlled and approved laboratory experiments. Animals were assigned to each experimental group such that the tumor luciferase activity was as similar as possible across all groups. Each group contained at least three mice.

### Reagents

IRDye 700DX-NHS ester was purchased from LI-COR Biosciences (Lincoln, Nevada, USA). Panitumumab, a fully humanized IgG2 mAb directed against EGFR, was purchased from Amgen (Thousand Oaks, California, USA). Anti-mouse PD-L1 (B7-H1) antibody (10F.9G2) and rat IgG2b (LTF-2; used as the control) were obtained from Bio X Cell (Lebanon, New Hampshire, USA).

### Cell culture

All cell lines were obtained from the American Type Culture Collection (Manassas, Virginia, USA). A431-luc-GFP cells (human epidermoid cancer cell) with genes encoding firefly luciferase and GFP,^19 20^ luciferase-expressing MC38 (murine colon cancer cell), LL/2 (murine Lewis lung carcinoma cell), TRAMP-C2 (murine prostate cancer cell), B16F0 (murine melanoma cell), and LL/2-Luc-GFP-PD-L1 cells (artificially overexpressed GFP-PD-L1) with genes encoding firefly luciferase,^21^ GFP, and mouse PD-L1 were cultured in RPMI-1640 (Thermo Fisher Scientific Inc, Waltham, Massachusetts, USA) supplemented with 10% fetal bovine serum and penicillin (100 IU/mL)–streptomycin (100 mg/mL) (Thermo Fisher Scientific Inc).

### Production of anti-PD-L1-F(ab’)_2_ and control-F(ab’)_2_ from anti-PD-L1-IgG and control-IgG, respectively

F(ab′)_2_ fragments of anti-mouse PD-L1 antibody (10F.9G2, anti-PD-L1-F(ab′)_2_) and control rat IgG2b (control-F(ab′)_2_) were produced by digesting the whole IgG antibody using immobilized ficin (Thermo Fisher Scientific Inc) in 10 mM citrate buffer with 4 mM cysteine and 5 mM EDTA (pH 6.0) at 37°C for 24 hours, as previously described.^21^ Next, F(ab′)_2_ was purified by high-performance liquid chromatography using phosphate-buffered saline (PBS) as the eluent.

### Conjugation of IR700 to panitumumab, anti-PD-L1- F(ab’)_2_, or control-F(ab’)_2_

Panitumumab (6.8 nmol) was incubated with IR700 NHS-ester (30.8 nmol, LI-COR Biosciences) in 0.1 mol/L Na_2_HPO_4_ (pH 8.6) at 25°C for 1 hour. Anti-PD-L1-F(ab′)_2_ or control-F(ab′)_2_ (9.1 nmol) was incubated with IR700 NHS-ester (63.7 nmol, LI-COR Biosciences) in 0.3 mL of 0.1 M Na_2_HPO_4_ (pH 8.6) at 25°C for 1 hour.^22^ The mixture was separated and purified with a Sephadex G50 column (PD-10; GE Healthcare, Piscataway, New Jersey, USA).^23^ The protein concentration was confirmed with a Coomassie Plus protein assay kit (Thermo Fisher Scientific Inc) by measuring absorption at 595 nm with spectroscopy (UV1900, Shimadzu, Japan).^24 25^ The IR700 concentration was measured via absorption at 689 nm with spectroscopy to confirm the number of fluorophore molecules conjugated to mAb (dye–mAb ratio).^26^ Bioactivity of the conjugated products was determined by testing its binding on LL/2-luc-GFP-PD-L1 cells. The cells (1×10^5^) were incubated with pan-700 (10 µg/mL) or anti-PD-L1-F(ab′)_2_-IR700 (10 µg/mL) in medium for 6 hours at 37°C. For confirming the binding specificity of the new conjugates, a competition assay was performed by adding excess untreated anti-PD-L1 antibody (1 µg). Cells were analyzed with flow cytometry (Gallios, BD Biosciences) using Kalulza2.1 software (BD Biosciences).

### Fluorescence microscopy

To detect the antigen-specific localization of IR700 conjugates, fluorescence microscopy was performed (A1Rsi; Nikon Instech, Tokyo, Japan). MC38-luc, LL/2-luc, and TRAMP C2-luc cells (2×10^4^) were seeded on glass-bottom dishes and incubated for 24 hours. Then, 10 µg/mL anti-PD-L1-F(ab′)_2_-IR700 was added to the culture medium, and cells were incubated at 37°C for 6 hours. Next, the cells were washed twice with PBS. SYTOX blue (final concentration: 2 µg/mL; Thermo Fisher Scientific Inc) was added 20 min before microscopic observation of ruptured cells. The cells were exposed to NIR light (20 J/cm^2^); serial microscopic images were captured.

### *In vitro* NIR-PIT

For EGFR-targeted-NIR-PIT, A431-luc-GFP cells (1×10^5^) were seeded onto 12-well plates and incubated with panitumumab-IR700 (pan-IR700; 10 µg/mL) containing medium for 12 hours at 37°C. For PD-L1-targeted NIR-PIT, MC-38-luc, LL/2-luc, Tramp-C2-luc, or B16F_0_ cells (1×10^5^) were seeded onto 12-well plates and incubated with anti-PD-L1-IR700 (10 µg/mL) containing medium for 12 hours at 37°C. After washing twice with PBS, the cells were irradiated using an NIR light-emitting diode at 670–710 nm wavelength (L690-66-60, Ushio-Epitex, Kyoto, Japan).^27^ The actual power density (mW/cm^2^) in the experiments was measured with an optical power meter (PM100; Thorlabs, Newton, New Jersey, USA).^28^

The photocytotoxic effects of NIR-PIT were measured by luciferase activity and flow cytometry with propidium iodide (PI, final concentration 2 µg/mL; Thermo Fisher Scientific Inc) staining. To monitor luciferase activity, 150 µg/mL D-luciferin-containing medium (Goryo Chemical, Sapporo, Japan) was administered to PBS-washed cells at 24 hours after NIR-PIT, and cells were analyzed with a plate reader to detect their bioluminescence (Powerscan4; BioTek, Winooski, Vermont, USA). For PI staining with flow cytometry to detect necrotic cells, the cells were stripped and dissociated with pipetting 1 hour after the treatment and washed twice with PBS. PI (final concentration 2 µg/mL) was added to the cell suspension, and cells were incubated at 25°C for 30 min before flow cytometry. PI fluorescence was evaluated using 1×10^4^ cells with FACS Calibur (Becton Dickinson, Franklin Lakes, New Jersey, USA).

### Animals and tumor models

All mice were purchased from Chubu Kagaku Shizai (Nagoya, Japan). During all experimental procedures, the mice were anesthetized with isoflurane. Approximately 10–15 week-old C57BL/6 mice were inoculated with MC38-luc, LL/2-luc, or TRAMP-C2-luc cells (2×10^6^) into the right, left, or both dorsa. Mice were shaved at the tumor sites for irradiation and imaging analysis. The largest longitudinal diameter (length) and transverse diameter (width) were measured with an external caliper. Tumor volumes based on caliper measurements were calculated using the following formula: tumor volume=length × width^2^ × 0.5. Body weight (BW) was measured on a scale. Mice were monitored daily, and tumor volumes were measured at least twice a week until the tumor (or any tumor for mice with multiple tumors) diameter reached 2 cm, whereupon the mice were euthanized with carbon dioxide.

### *In vivo* bioluminescence imaging (BLI)

For BLI, D-luciferin (15 mg/mL, 200 µL) was injected intraperitoneally, and the mice were captured on a bioluminescence imager (IVIS, PerkinElmer) to measure the luciferase activity. Regions of interest were set on whole tumors to quantify luciferase activities.^29^

### *In vivo* IR700-fluorescence imaging

IR700-fluorescence was detected before and after the therapy using a fluorescence imager (Pearl Imager, LI-COR Biosciences).^30^

### *In vivo* PD-L1-targeted-NIR-PIT

PD-L1-targeted NIR-PIT on the tumor was performed at 4 days after tumor inoculation. The following day, mice were injected with 100 µg anti-PD-L1-F(ab′)_2_-IR700 or control-F(ab′)_2_-IR700 and irradiated with NIR light at 75 J/cm^2^, unless otherwise specified, to the right tumor.

### Analysis of tumor-infiltrating, splenic and peripheral blood lymphocytes

To characterize the systemic effect of anti-PD-L1-F(ab′)_2_-IR700 administration on lymphocytes, 100 µg anti-PD-L1 IgG or anti-PD-L1-F(ab′)_2_ was injected intravenously into mice; spleen lymphocytes were analyzed the next day. To test the effects of NIR-PIT with anti-PD-L1-F(ab′)_2_-IR700 on various tumor lymphocytes, blood and the spleen were harvested at the indicated time after NIR-PIT. The cells were stained with antibodies against CD3e (145–2 C11), CD8a (53–6.7), CD25 (3C7), NK1.1 (PK136), CD11c (N418), CD11b (M1/70), Ly-6G (1A8-Ly6g), CD45 (30-F11), F4/80 (BM8), and CD69 (H1.2F3) for 1 hour. All antibodies were purchased from eBioscience (Thermo Fisher Scientific Inc). Foxp3 was stained with Foxp3/Transcription Factor Fixation/Permeabilization Concentrate and Diluent (Thermo Fisher Scientific Inc) and antibodies against Foxp3 (FJK-16s). Interferon-gamma (IFN-γ) and interleukin-2 (IL-2) were stained with Fixation/Permeabilization Concentrate and Diluent (Thermo Fisher Scientific Inc) and antibodies against IFN-γ (XMG1.2) and IL-2 (JES6-5H4), respectively, according to the manufacturer’s instructions. The stained cells were analyzed by flow cytometry (FACS CantoII, BD Biosciences), and the data were analyzed with FlowJo software (FlowJo LLC, Ashland, Oregon, USA). All FACS markers are indicated in the online supplemental material.

10.1136/jitc-2021-003036.supp1 Supplementary data

### Serum and intratumoral cytokine analysis

Tumor inoculation and the treatment were performed as described earlier. Sera were serially collected from the mice before, and at 6 and 24 hours after PD-L1-targeted NIR-PIT on MC38-luc tumors. Tumors were harvested and homogenized in 1 mL PBS supplemented with protease inhibitors (cOmplete Tablets; Sigma-Aldrich, St. Louis, Missouri, USA); then, the solution was passed through a filter (Cell Strainer 70 µm Nylon; Corning, Corning, New York, USA). Concentrations of various cytokines and chemokines in the samples were analyzed with Mouse Cytokine Array/Chemokine Array from Eve Technologies (Calgary, Canada).

### Damage-associated molecular pattern (DAMP) analysis

MC38 (5×10^5^) and MC38-luc (1×10^5^) cells for ATP and high mobility group box protein 1 (HMGB-1) expression quantification, respectively, were seeded onto 12-well plates and incubated with 10 µg/mL anti-PD-L1-IR700 for 12 hours at 37°C. After replacing the medium with PBS, the cells were irradiated with 128 J/cm^2^ NIR light. ATP expression was quantified by FF2000 ENLITEN ATP Assay System (Promega, Madison, Wisconsin, USA) and HMGB1 expression by ELISA (ARG81310; Arigo Biolaboratories, Hsinchu, Taiwan) at 1-hour post-treatment.

### Quantification of PD-L1 expression

In vitro or in vivo PD-L1 expression was analyzed with the treated cells or mice tumors, respectively. For in vitro analysis, MC38-luc cells (1×10^4^) were treated with PD-L1-targeted NIR-PIT, and then, the treated cells were collected 5 days after NIR-PIT. For in vivo analysis, tumor cells were collected at 7 days after treatment. The cells were stained with antibodies against PD-L1 (1–111A) and analyzed using a flow cytometer (Gallios, BD Biosciences). Data were analyzed with Kaluza V.2.1 software (BD Biosciences).

### Mass cytometry (CyTOF)

To test the systemic effects of PD-L1-targeted NIR-PIT on various lymphocytes, the peripheral blood was harvested at the indicated time after NIR-PIT. The cells were stained with Maxpar Mouse Spleen/Lymph node phenotyping panel kit (Fluidigm, Tokyo, Japan). Samples were analyzed by Helios mass cytometer, and data were analyzed with FlowJo software (FlowJo LLC).

### Immune depletion of natural killer (NK) and CD8 T cells and neutralization of IFN-γ *in vivo*

Anti-NK1.1 (PK136) or anti-CD8a (2.43) depleting antibody, or anti-IFN-γ (XMG1.2) neutralizing antibody was injected intraperitoneally every 2 days starting 2 days before NIR-PIT at the doses of 25, 50, and 100 µg, respectively, until euthanasia. All the antibodies were purchased from Bio X Cell.

### Statistics

Data are expressed as means±SEM from a minimum of four experiments, unless otherwise indicated. Statistical analyses were performed using GraphPad Prism (GraphPad Software, San Diego, California, USA). The cumulative probability of survival, defined as the tumor diameter failing to reach 2 cm, was estimated in each group with the Kaplan-Meier survival curve analysis, and the results were compared via the log-rank test and Wilcoxon test. For two-group comparisons, an unpaired t-test was performed. For multiple-group comparisons, a one-way analysis of variance with Tukey’s test was used. P<0.05 indicated statistically significant differences.

## Results

### Production and evaluation of Fc-deficient anti-PD-L1-F(ab’)_2_-IR700 *in vivo*

To avert Fc-mediated in vivo complement-dependent cytotoxicity and antibody-dependent cellular cytotoxicity, F(ab′)_2_ fragments were produced from anti-PD-L1 antibody (anti-PD-L1-F(ab′)_2_) and control IgG antibody (control-F(ab′)_2_), and purified F(ab′)_2_ fragments were conjugated with the IR700 dye (anti-PD-L1-F(ab′)_2_-IR700 and control-F(ab′)_2_-IR700, respectively) (online supplemental figure 1A). The binding of anti-PD-L1-F(ab′)_2_-IR700 on PD-L1-overexpressing LL/2 (LL/2-luc-GFP-PD-L1) cells was blocked with excess anti-PD-L1-IgG, suggesting that the produced anti-PD-L1-F(ab′)_2_-IR700 specifically bound to PD-L1 (online supplemental figure 1B).

**Figure 1:**
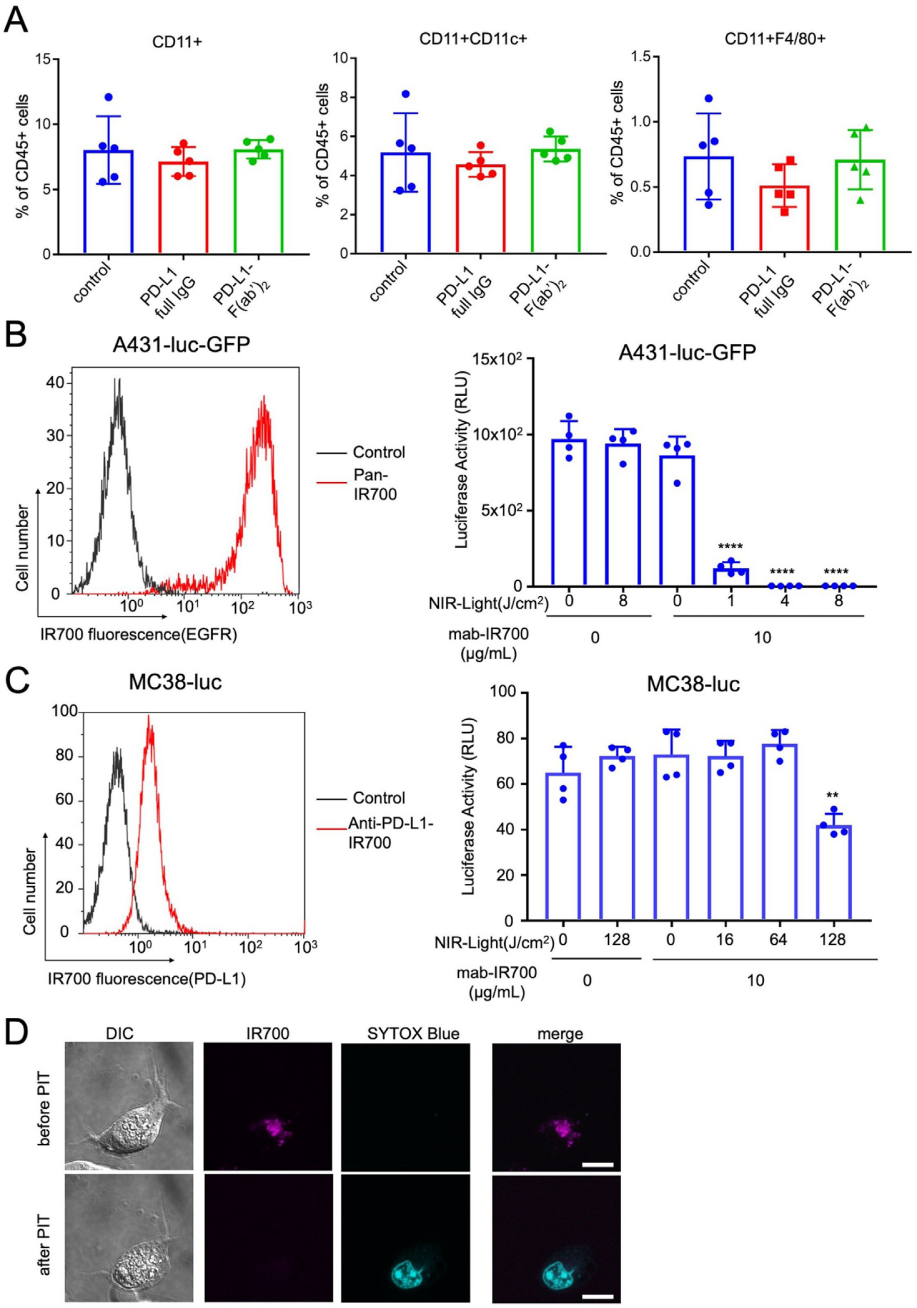
Production of Fc-deficient anti-PD-L1- F(ab′)_2_–IR700 and evaluation *in vivo*, and evaluation of *in vitro* NIR-PIT with anti-PD-L1- F(ab′)_2_–IR700 on various murine tumor cells. (A) Intravenously injected anti-PD-L1-IgG (100 μg) or anti-PD-L1- F(ab’)_2_ (100 μg) did not alter the percentage of antigen-presenting cells (APCs) in the spleen CD45-positive cells 1 day after administration. Data are the mean±SEM (n=5; p≥0.05, Student’s t-test). (B) (Left) EGFR expression was evaluated on high EGFR-expressed A431-luc-GFP cells with pan-IR700 by flow cytometry. (Right) *In vitro* EGFR-targeted NIR-PIT with pan-IR700 was measured by luciferase activity, which decreased in a NIR light dose-dependent manner. Data are the mean±SEM (n=4, ****p<0.0001, Student’s t-test). With a light dose of 4 (J/cm^2^), *in vitro* EGFR-targeted NIR-PIT destroyed all A431-luc-GFP cells. (C) (Left) PD-L1-expression was evaluated on MC38-luc with PD-L1-F(ab’)_2_-IR700 by flow cytometry. (Right) *In vitro* PD-L1-targeted NIR-PIT with PD-L1-F(ab’)_2_-IR700 on MC-38-luc was measured by luciferase activity, which decreased in a NIR light dose-dependent manner. Data are the mean±SEM (n=4, **p<0.01, Student’s t-test). Even with a light dose of 128 (J/cm^2^), *in vitro* PD-L1-targeted NIR-PIT destroyed only 40%–50% of MC38-luc cells. *In vitro* PD-L1-targeted NIR-PIT with PD-L1-F(ab’)_2_-IR700 on other murine tumor cells (LL/2-luc, Tramp-C2-luc, B16F_0_) was also evaluated (online supplemental figure 2B). (D) Microscopic observations before and after PD-L1-targeted NIR-PIT. MC38-luc cells were incubated with anti-PD-L1-F(ab’)_2_-IR700 for 6 hours and observed with a fluorescence microscope before and after NIR light irradiation (20 J/cm^2^). Photo-induced necrotic cell death was observed after exposure to NIR light at 20 min after NIR-PIT (scale bar, 10 µm). Other murine tumor cells (LL/2-luc, Tramp-C2-luc) was also observed as the same way as MC38-luc (online supplemental figure 2C). EGFR, epidermal growth factor receptor; NIR-PIT, near-infrared photoimmunotherapy; PD-L1, programmed death-ligand 1.

We evaluated the systemic effects of administering anti-PD-L1-IgG or anti-PD-L1-F(ab′)_2_ to mice by analyzing the splenic antigen-presenting cells (APCs) 1 day after administration. No significant change in the percentage of APCs was observed among the splenic CD45-positive cells (figure 1A). However, administration of either anti-PD-L1-IgG or anti-PD-L1-F(ab′)_2_ decreased PD-L1 expression by splenic APCs. The decrease in the PD-L1-positive population following treatment with anti-PD-L1-F(ab′)_2_ was lesser than that observed following anti-PD-L1-IgG treatment (online supplemental figure 1C). These data indicated that applying anti-PD-L1-F(ab′)_2_ in vivo led to a slight decrease in PD-L1-expressing APCs; however, there were no remarkable effects on splenic APCs.

### NIR-PIT with Fc-deficient anti-PD-L1-F(ab′)_2_-IR700 induces limited necrotic cell death of mouse tumor cells *in vitro* compared with EGFR-targeted NIR-PIT

To compare the expression of target cell-surface proteins, we analyzed EGFR expression using flow cytometry on A431-luc-GFP cells and PD-L1 expression on MC38-luc, LL/2-luc, Tramp-C2-luc, and B16F_0_-luc cells (figure 1B,C and online supplemental figure 2A). PD-L1 on MC38-luc cells was expressed approximately 100 times lesser than the overexpressed EGFR on A431-luc-GFP cells, as evaluated using IR700-fluorescence on the cell surface. The same PD-L1 expression level was also confirmed on LL/2-luc, Tramp-C2-luc, and B16F_0_-luc cells. Although PD-L1 expression on murine tumor cells was low, its expression was universal. Furthermore, the *in vitro* cytotoxic effect of EGFR-targeted NIR-PIT on A431-luc-GFP cells with pan-IR700 was higher than that of PD-L1-targeted NIR-PIT on MC38-luc cells. EGFR-targeted NIR-PIT on A431-luc-GFP cells with pan-IR700 could kill almost all cells with a mild NIR irradiation at 4 J/cm^2^. PD-L1-targeted NIR-PIT on MC38-luc cells could kill approximately 50% cells with irradiation at 128 J/cm^2^. The efficacy of PD-L1-targeted NIR-PIT on other murine tumor cells (LL/2-luc, Tramp-C2-luc, and B16F_0_-luc) was similar to that on MC38-luc cells (online supplemental figure 2B).

*In vitro* PD-L1-targeted NIR-PIT on MC38-luc cells induced cellular swelling and bleb formation (figure 1D) as confirmed by fluorescence microscopy and staining with the cytotoxicity marker SYTOX Blue. Other murine tumor cells were also destroyed (online supplemental figure 2C).

Taken together, *in vitro* PD-L1-targeted NIR-PIT could induce a cytotoxic effect on murine tumor cells; however, its efficacy was limited.

### PD-L1-targeted NIR-PIT induces unexpected remarkable tumor regression *in vivo*

**Figure 2:**
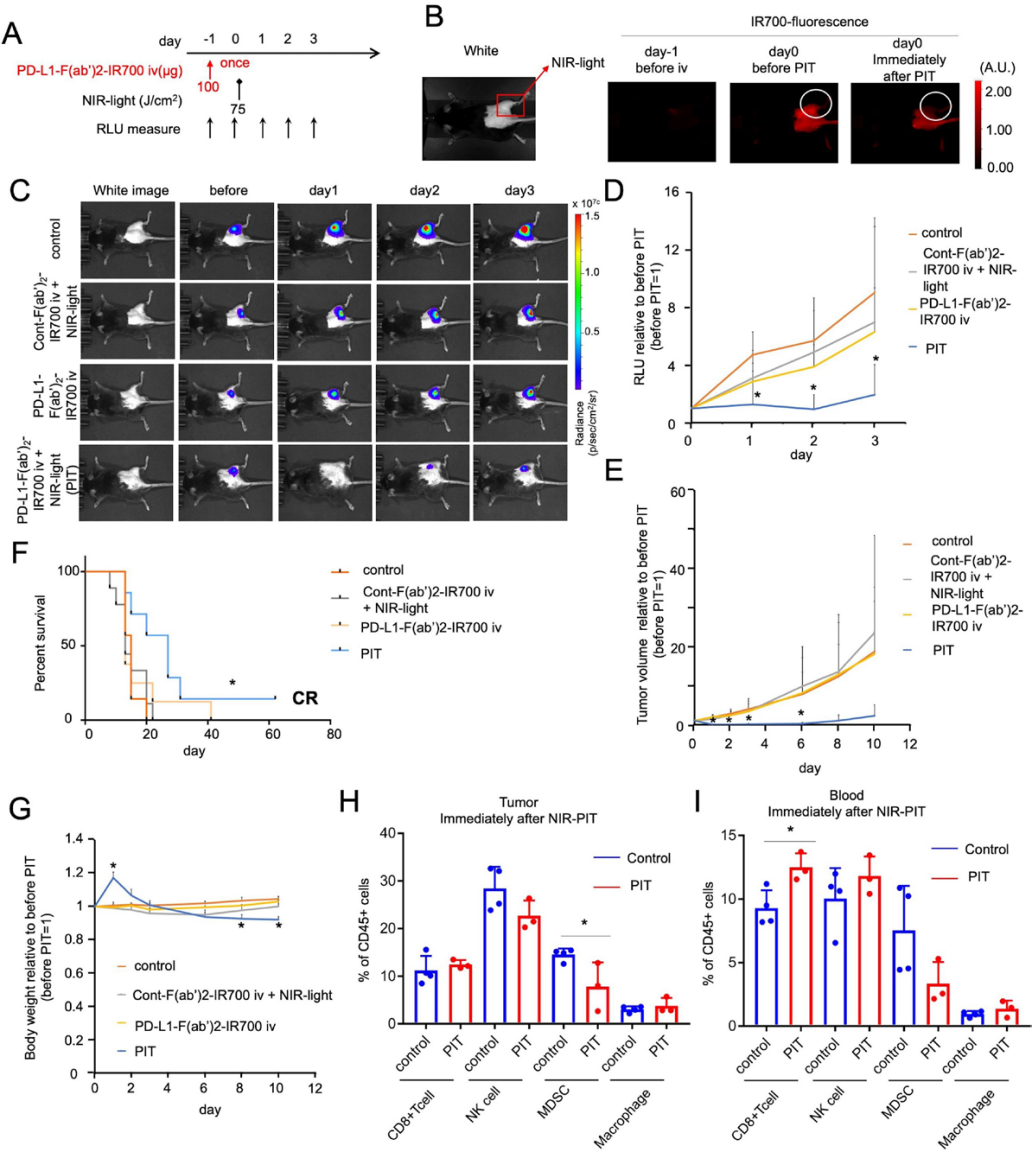
*In vivo* NIR-PIT targeting PD-L1 induces unexpected remarkable regression of treated tumors. (A) PD-L1-targeted NIR-PIT regimen involving anti-PD-L1-F(ab′)_2_-IR700 injection, NIR light-exposure, and BLI evaluation are shown. The anti-PD-L1-F(ab′)_2_-IR700 injection was performed only once at 1 day before light exposure. (B) Accumulation of IR700-fluorescence in anti-PD-L1-F(ab′)_2_-IR700 at 1 d after the injection, which was attenuated immediately after NIR-PIT. (C) In vivo bioluminescence imaging (BLI) of MC38-luc tumor-bearing mice along with the treatment. Relative light unit (RLU) measurements decreased only in the NIR-PIT group (anti-PD-L1-F(ab′)_2_-IR700 and NIR light irradiation), which meant tumor luciferase activities was decreased after the therapy. (D) Quantitative RLU showed a significant decrease in PD-L1-targeted NIR-PIT-treated tumors (n=7–9 in each group) (PIT group vs control group at day 1: *p<0.01; PIT group vs control group at day 2: *p<0.01; PIT group vs cont-F(ab′)_2_ group at day 2: *p<0.05; PIT group vs control group at day 3: *p<0.05, Tukey’s test with ANOVA). (E) Measurement of tumor volume ratio (before NIR-PIT=1) was evaluated. PD-L1-targeted NIR-PIT elicited significant reductions in the tumor volume ratio whereas neither PD-L1-F(ab′)_2_-IR700 injection, nor NIR light irradiation alone, nor cont-F(ab′)_2_-IR700 with NIR light irradiation showed increasing tumor reduction day by day (n=7–9 in each group) (PIT group vs all other groups at day 1: *p<0.0001; PIT group vs control group at day 2: *p<0.0001; (PIT group vs all other groups at day 1: *p<0.0001; PIT group vs control group at day 2: *p<0.0001; PIT group vs cont-F(ab′)_2_ group at day 2: *p<0.0001; PIT group vs APC only group at day 2: *p<0.0001; PIT group vs control group at day 3: *p<0.001; PIT group vs cont-F(ab′)_2_ group at day 3: *p<0.001; PIT group vs APC only group at day 3: *p<0.01; PIT group vs control group at day 6: *p<0.01; PIT group vs cont-F(ab′)_2_ group at day 6: *p<0.05; PIT group vs APC only group at day 6: *p<0.05; PIT group vs cont-F(ab′)_2_ group at day 8: *p<0.05; Tukey’s test with two-way repeated measures ANOVA). PD-L1-targeted NIR-PIT on other types of murine tumors (Tramp-C2, LL/2) also demonstrated significant regression (online supplemental figure 3). (F) PD-L1-targeted NIR-PIT led to prolonged survival in tumor-bearing mice (n=7–9 in each group) (*p=0.0119 < 0.05, log-rank test). One mouse displayed a complete response and totally cured. (G) The body weight (BW) of treated mice is shown. The BW of mice not receiving PD-L1-targeted NIR-PIT gradually increased due to tumor growth. In contrast, the BW of PIT group mice showed transient increase due to edema in and around treated tumors and then a decrease was observed. (n=7–9 in each group, PIT group vs control group at day 1: *p<0.01; PIT group vs cont-F(ab′)_2_ with NIR light group at day 1: *p<0.001; PIT group vs PD-L1-F(ab’)_2_-IR700 intravenous injection group at day 1: *p<0.001; PIT group vs control group at day 8: *p<0.05; PIT group vs control group at day 10: *p<0.01; PIT group vs PD-L1-F(ab′)_2_-IR700 intravenous injection group at day 10: *p<0.05; Tukey’s test with ANOVA). (H) Analysis of the percentage of CD8 + T cells (CD3 +CD8+), NK cells (CD3-NK1.1+), MDSCs (CD11b+Gr1+), and macrophages (CD11b+F4/80+) within CD45 + cells in the tumors immediately after PD-L1-targeted NIR-PIT (n=3–4; *p<0.05, Student’s t-test). No significant difference was detected in CD8 +T cells, NK cells, and macrophages. MDSCs were significantly depleted in PD-L1-targeted NIR-PIT-treated tumors. (I) Analysis of the percentage of CD8 + T cells, NK cells, MDSCs, and macrophages relative to CD45 + cells in the blood immediately after PD-L1-targeted NIR-PIT (n=3–4; *p<0.05, Student’s t-test). No significant difference was detected in NK cells, and MDSCs, and macrophages. CD8 + T cells were significantly augmented in PD-L1-targeted NIR-PIT treated peripheral blood. ANOVA, analysis of variance; BW, body weight; MDSCs, myeloid-derived suppressor cells; NK, natural killer; PD-L1, programmed death-ligand 1; PIT, photoimmunotherapy.

We evaluated the effect of local PD-L1-targeted NIR-PIT against MC38-luc flank tumors *in vivo* (figure 2A). Anti-PD-L1-F(ab′)_2_-IR700 was injected in mice only once, with the NIR light irradiation (75 J/cm^2^) performed 1 day after drug administration (figure 2A). We expected the light dose to kill less than 40% of the tumor cells *in vitro* (figure 1C). The experimental group of mice with MC38-luc tumors received anti-PD-L1-F(ab′)_2_-IR700 injection, followed by NIR light exposure (referred to as the PIT group). This group showed an increase in fluorescence due to the accumulation of anti-PD-L1-F(ab′)_2_-IR700 inside the tumor 1 day after injection, and this increase was attenuated immediately after NIR light irradiation (figure 2B). The PIT group showed reduced tumor luciferase activity, as indicated via BLI on day 1, compared with that in the three control groups: untreated mice (control), mice receiving control F(ab′)_2_-IR700 with NIR light, and mice receiving anti-PD-L1-F(ab′)_2_-IR700 once without irradiation (figure 2C). Quantification of luciferase activity revealed significant decreases (4–5 fold decrease) in relative light units in the PIT group at 3 days after treatment, whereas the other groups showed a gradual increase in relative units with tumor growth (figure 2D). Consistent with the BLI results, tumor volumes in the PIT group were 15–20 times lower than those in the other groups (figure 2E). Mouse survival was significantly prolonged in the NIR-PIT groups compared with that in the control groups (*p<0.05 (0.0119); log-rank test; figure 2F). Similar findings were obtained with both LL/2-luc and TRAMP C2-luc syngeneic tumors (LL/2-luc, online supplemental figure 3A–D; TRAMP C2-luc, online supplemental figure 3E–H). BW of mice that did not receive PD-L1-targeted NIR-PIT gradually increased due to tumor growth. Contrastingly, BW in the PIT group increased transiently due to edema, suggesting the induction of a strong immune reaction and subsequently decreased to the same level as that in other groups as the edema resolved. On day 8, the PIT group showed significantly lower BW than the control group due tumor regression (*p<0.05 (0.0128), PIT vs control group at day 8; figure 2G).

Analysis of tumor-infiltrating lymphocytes immediately after the treatment revealed that MDSC (CD11b^+^Gr1^+^) counts decreased significantly, but no significant changes were detected in the counts of CD8 T (CD3^+^CD8^+^) cells, NK (CD3^−^NK1.1^+^) cells, and macrophages (CD11b^+^ F4/80^+^) (figure 2H). In the peripheral blood, a significant increase was observed in CD8^+^ T cell count, but not in that of the other populations (figure 2I). PD-L1-targeted NIR-PIT did not affect the antitumor effector cells, with only intratumoral MDSC depletion.

Collectively, these data demonstrated the remarkable antitumor effect of PD-L1-targeted NIR-PIT *in vivo*, an unexpected result compared with the *in vitro* results. Lymphocyte analysis revealed that the treatment successfully depleted only intratumoral MDSCs, suggesting the involvement of an antitumor immune response in this unexpected effectivenes*s in vivo*.

### PD-L1-targeted NIR-PIT induces rapid activation of tumor-infiltrating CD8 T and NK cells *in vivo*

**Figure 3:**
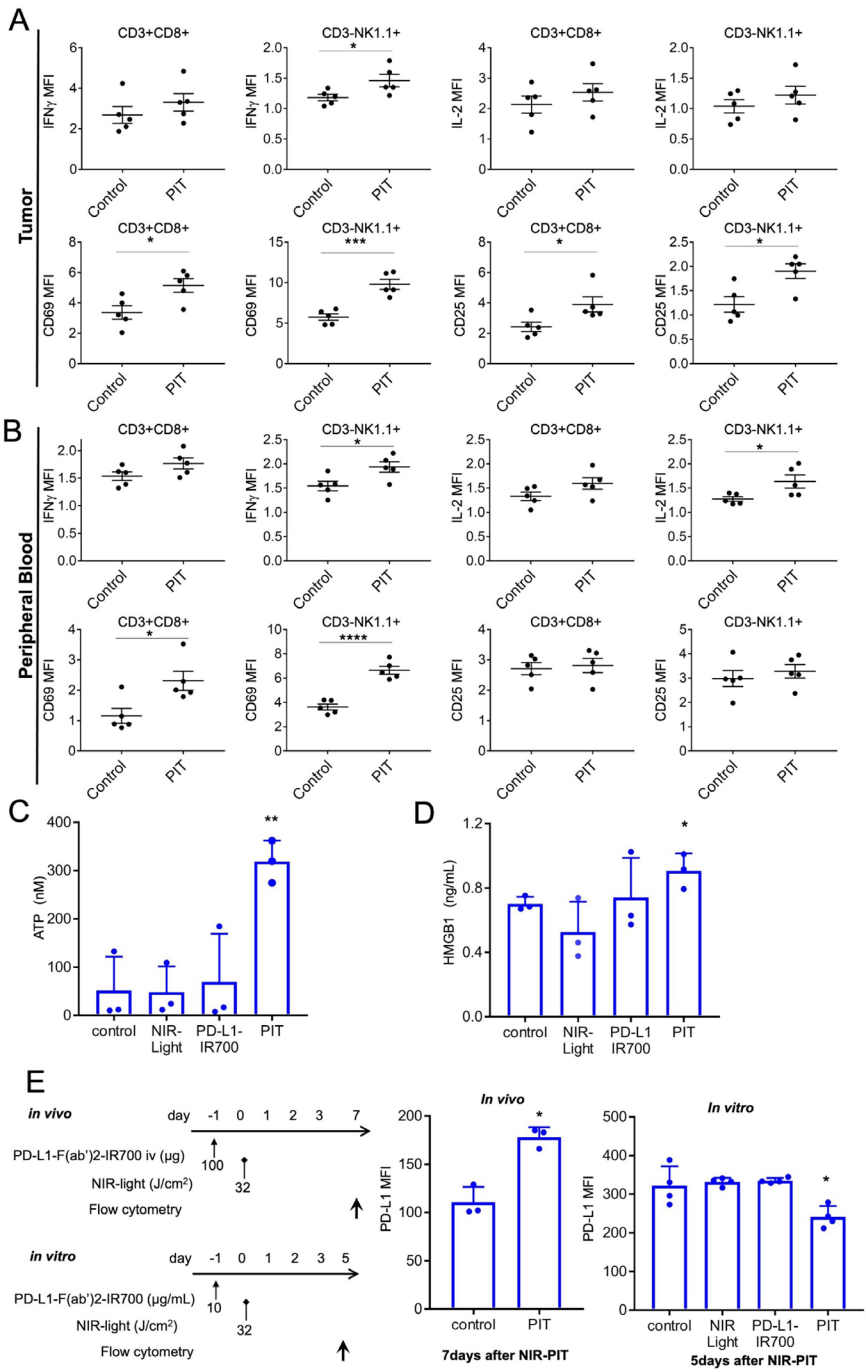
*In vivo* PD-L1-targeted NIR-PIT induces rapid activation of tumor-infiltrating CD8 T and NK cells. (A) Cytotoxic action of CD8 T and NK cells infiltrating in MC38-luc tumors was examined by flow cytometry with or without PD-L1-targeted NIR-PIT. CD8^+^ T and NK cells collected 1.5 hours after NIR-PIT were producing IFN-γ and had CD69, CD25 exposed on the cell surface, whereas the cells from nontreated tumors did not (n=5; *p<0.05, ***p<0.001, Student’s t-test). (B) Cytotoxic action of CD8^+^ T and NK cells in peripheral blood was analyzed by flow cytometry with or without PD-L1-targeted NIR-PIT. CD8^+^ T and NK cells collected 1.5 hours after NIR-PIT were producing IFN-γ and had CD69 exposed on the cell surface, whereas the cells from nontreated tumors did not (n=5; *p<0.05, **p < 0.01, ****p<0.0001, Student’s t-test). (C) NIR-PIT induces a rapid release of adenosine triphosphate (ATP). MC38 cells were incubated with PD-L1-IR700 (10 µg/mL) and exposed to NIR light. Extracellular ATP concentrations were measured with luciferase activity. Data are means±SEMs (n=3, **p<0.01, Student’s t-test). (D) NIR-PIT induces a rapid release of DAMPs. MC38-luc cells were incubated with PD-L1-F(ab’)_2_-IR700 (10 µg/mL), then NIR light-irradiated and supernatants were analyzed with ELISA. Data are means±SEMs (n=3, *p<0.05, Student’s t-test). (E) Increased PD-L1-expression was observed in MC38-luc tumors in vivo 7 days after 32 J/cm^2^ PD-L1-targeted NIR-PIT (n=3, *p<0.05, Student’s t-test). PD-L1-targeted NIR-PIT could augment PD-L1 expression on the treated tumor. However, PD-L1 expression was decreased in vitro 5 days after 32 J/cm^2^ PD-L1-targeted NIR-PIT (n=4, *p<0.05, Student’s t-test). DAMPs, damage-associated molecular patterns; IFN-γ, interferon-gamma; NIR-PIT, near-infrared photoimmunotherapy; NK, natural killer; PD-L1, programmed death-ligand 1.

We evaluated whether PD-L1-targeted NIR-PIT activated CD8 T and NK cells in the tumor or peripheral blood. As early as 1.5 hours after the treatment, intratumoral CD8 T and NK cells showed augmented IFN-γ and IL-2 expression, indicating their activation and tumor cell killing (figure 3A). Upregulation of CD69 and CD25 expression was also detected on these effector cells (figure 3A). CD8 T and NK cells in the peripheral blood were also activated at 1.5 hours after the treatment (figure 3B). However, administering anti-PD-L1-F(ab′)_2_-IR700 alone did not cause the aforementioned enhancement either in the tumor or peripheral blood (online supplemental figure 4A,B). Tregs in the tumor, blood, and spleen were not affected by PD-L1-targeted NIR-PIT (online supplemental figure 4C).

Next, we tested for the release of DAMPs, which induced immunogenic cell death after the therapy. As NIR-PIT is based on photo-induced necrosis, PD-L1-targeted NIR-PIT also increased ATP and HMGB1 levels *in vitro* (figure 3C,D). This suggested that PD-L1-targeted NIR-PIT also enhanced innate immune responses.

To determine changes in blood lymphocyte counts, CyTOF analysis was performed at 6 and 24 hours after therapy (online supplemental figure 5A). We found significant decreases in the counts of B, CD4 T, and CD8 T cells at both 6 and 24 hours after NIR-PIT and significant increases in the counts of MDSCs (6 hours after NIR-PIT), macrophages/monocytes (24 hours after NIR-PIT), dendritic cells (6 and 24 hours after NIR-PIT; online supplemental figure 5B). These results suggested that the localized PD-L1-targeted NIR-PIT induced a systemic antitumor immune reaction, resulting in recruiting immune cells to the tumor site. Blood biomarker data could be used to confirm the therapeutic effects of PD-L1-targeted NIR-PIT.

With these strong immune reactions, we hypothesized that PD-L1-targeted NIR-PIT could alter intratumoral PD-L1 expression. As immune checkpoint inhibitors (ICIs) evidently augment antitumor effects via the PD-1/PD-L1 signaling axis, along with enhanced expression of their target PD-L1,^31 32^ altering PD-L1 expression is considered significant.^33^ PD-L1 is reportedly inducible by inflammatory cytokines, especially IFNs.^34^ Since analysis of the intratumoral effector cells revealed that IFN-γ expression was elevated (figure 3A), PD-L1-targeted NIR-PIT might edit the tumor cell PD-L1 profile. PD-L1 expression was augmented in vivo 7 days after treatment, whereas it was downregulated in vitro (figure 3E). As in vitro PD-L1-targeted NIR-PIT selectively destroyed the population with high PD-L1 expression among the cultured cells, cells with a lower PD-L1 expression survived. These data indicated that PD-L1-targeted NIR-PIT could enhance PD-L1 expression in response to immune reactions, which would be advantageous for repeated PD-L1-targeted NIR-PIT or additional anti-PD-1/PD-L1 therapy.

To summarize, these findings suggested that after PD-L1-targeted NIR-PIT, both direct photocytotoxicity and antitumor ‘photoimmuno’ reactions of the effector cells enhanced each other. We speculate that the unexpected effects of PD-L1-targeted NIR-PIT in vivo can be attributed to enhancing antitumor immunity.

### PD-L1-targeted NIR-PIT induces a systemic and intratumoral cytokine storm

We investigated changes in both serum and intratumoral cytokine and chemokine concentrations after PD-L1-targeted NIR-PIT. The levels of various cytokines and chemokines increased in the serum at 6 hours (online supplemental figure 6A) and in the tumors 24 hours after the therapy (online supplemental figure 6B). Only local intratumoral PD-L1-targeted NIR-PIT could induce systemic inflammation, which resembled a clinical cytokine storm.^35 36^

### Therapeutic effects of PD-L1-targeted NIR-PIT extend to distant untreated tumors, inducing abscopal effects

**Figure 4:**
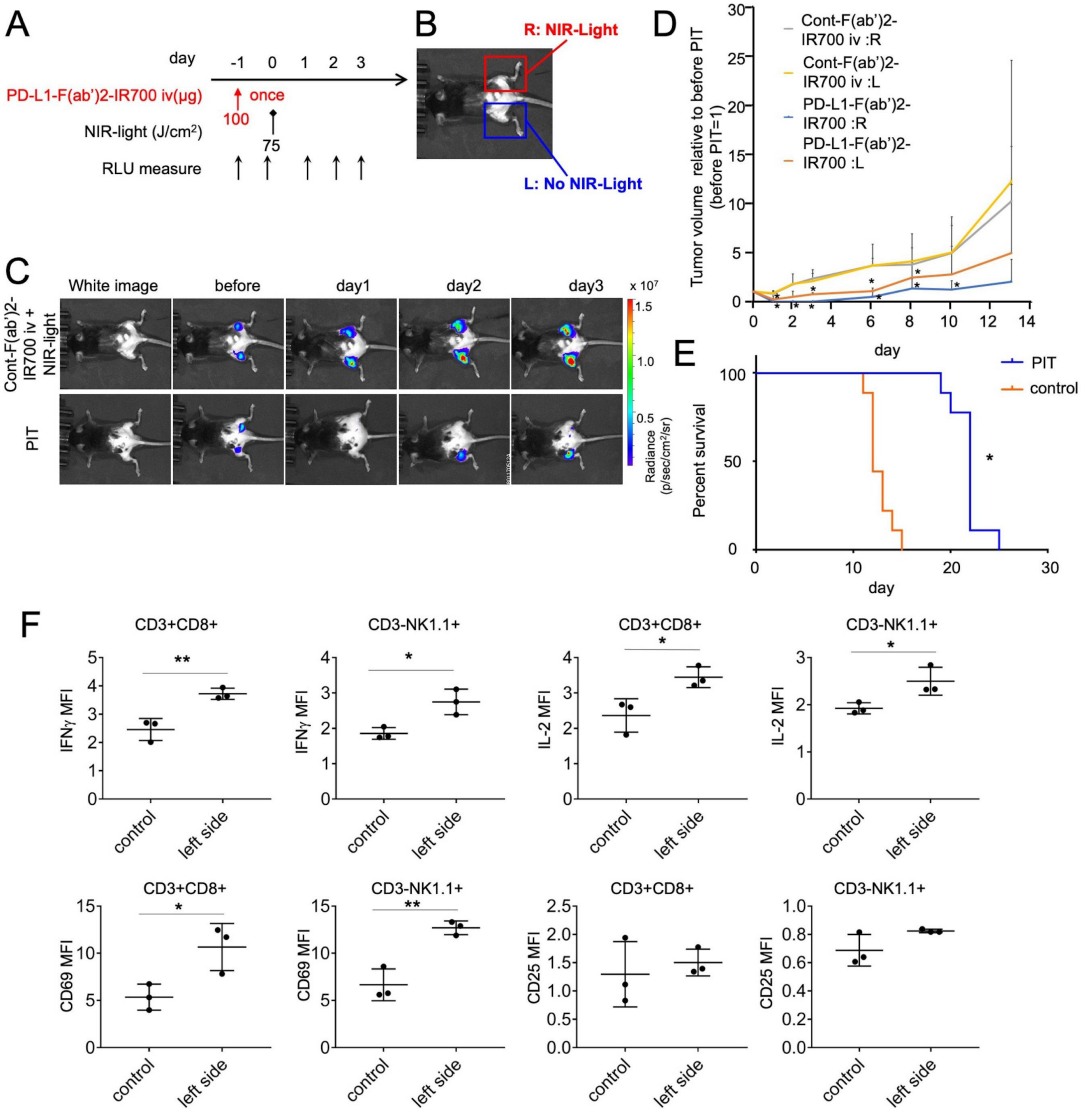
Therapeutic effects of PD-L1-targeted NIR-PIT could extend to distant non-NIR light irradiated tumors, inducing abscopal effects. (A) PD-L1-targeted NIR-PIT regimen is shown. (B) Mice with bilateral flank tumors were either injected with control F(ab′)_2_–IR700 or PD-L1-F(ab’)_2_-IR700, followed by NIR light irradiation of only the right tumor. One day after PD-L1-F(ab′)_2_-IR700 administration, the tumor on the right was irradiated with NIR light. (C) In vivo BLI of bilateral flank tumor model is demonstrated. In vivo BLI showed changes in bioluminescence signals in both tumors in response to PD-L1-targeted NIR-PIT on the right tumor. However, NIR light irradiation with control F(ab′)_2_–IR700 induced no decrease in either tumor. Before NIR-PIT, tumors were approximately the same size and exhibited similar bioluminescence. (D) Tumor volume ratio (as defined before NIR-PIT=1). PD-L1-targeted NIR-PIT introduced on day 0 led to significant tumor-volume reductions in the treated right and untreated left side (n=4–6 in each group) (PD-L1:R vs Cont:R and L at day 1:*p<0.001; PD-L1:L vs Cont:R and L at day 1:*p<0.01; PD-L1:R vs Cont:R and L at day 2:*p<0.01; PD-L1:L vs Cont:R and L at day 2:*p<0.05;PD-L1:R vs Cont:R and L at day 3:*p<0.01; PD-L1:L vs Cont:R and L at day 3:*p<0.05;PD-L1:R vs Cont:R and L at day 6:*p<0.001; PD-L1:L vs Cont:R and L at day 6:*p<0.01, PD-L1:R vs Cont:R and L at day 8:*p<0.01; PD-L1:L vs Cont:R and L at day 8:*p<0.05, PD-L1:R vs Cont:R and L at day 10:*p<0.01; Tukey’s test with two-way repeated measures ANOVA). (E) PD-L1-targeted NIR-PIT led to prolonged survival in bilateral tumor-bearing mice (n=9 in each group) (*p<0.0001, log-rank test). (F) CD8 T and NK cells collected from non-irradiated left dorsal tumors in mice receiving PD-L1-targeted NIR-PIT on the right dorsal tumors were analyzed for their expression of activation markers at 6 hours after the treatment (n=3; *p<0.05, **p<0.01, Student’s t-test). ANOVA, analysis of variance; NIR-PIT, near-infrared photoimmunotherapy; NK, natural killer; PD-L1, programmed death-ligand 1.

We hypothesized that the rapid antitumor immune activation and regression of the PIT-treated tumor would enable activation of antitumor effects resulting in the attacking of other tumor locations distant from the NIR-PIT-treated lesions. PD-L1-targeted NIR-PIT was performed only on the right side tumors in mice bearing bilateral MC38-luc flank tumors, covering the left side tumors (Figure 4A,B). BLI showed that luciferase activities decreased in the irradiated right-side tumor and in the non-irradiated left side tumor (figure 4C). Quantitative analysis of luciferase activities indicated a greater decrease in the NIR-PIT group than in the control group (online supplemental figure 7); the tumor volumes on either side also decreased significantly in the NIR-PIT group compared with that in the control group (figure 4D). We treated mice with bilateral tumor implantation with NIR-PIT and evaluated the prognosis until the tumor on either side reaches up to 2 cm. The prognosis of the NIR-PIT group was longer than that of the control-F(ab′)_2_-IR700-treated group (n=9 in each group, p<0.0001, log-rank test) (figure 4E). Moreover, the growth of tumor inoculated on the contralateral side of PIT-treated tumor at 1 day after the localized PD-L1-targeted NIR-PIT was inhibited compared with that in animals treated with control-F(ab′)2–IR700 and NIR light irradiation (online supplemental figure 8). This suggested that the antitumor effects were immune-mediated and immunological memory worked.

These data suggested that localized PD-L1-targeted NIR-PIT exerted antitumor effects on distant tumors, termed as abscopal effects.

### Effector CD8 T and NK cells in the non-irradiated tumor are also activated on PD-L1-targeted NIR-PIT

We next measured whether the non-irradiated left tumor contained activated CD8 T and NK cells after PD-L1-targeted NIR-PIT on the contralateral tumor. At 6 hours after therapy on the right side tumor, there was an increase in the levels of IFN-γ and IL-2 in intratumoral CD8 T or NK cells. Upregulated expression of the CD8 T and NK cell activation marker CD69 was also detected within the non-irradiated left tumor (figure 4F). Thus, the immune responses triggered by PD-L1-targeted NIR-PIT on the right side induced similar changes in untreated tumors located on the opposite side; as this effect was induced by NIR light, we termed it the ‘photo-abscopal effect.’

### Antitumor effects of PD-L1-targeted NIR-PIT depend partially on CD8 T and NK cells and IFN-γ production

**Figure 5:**
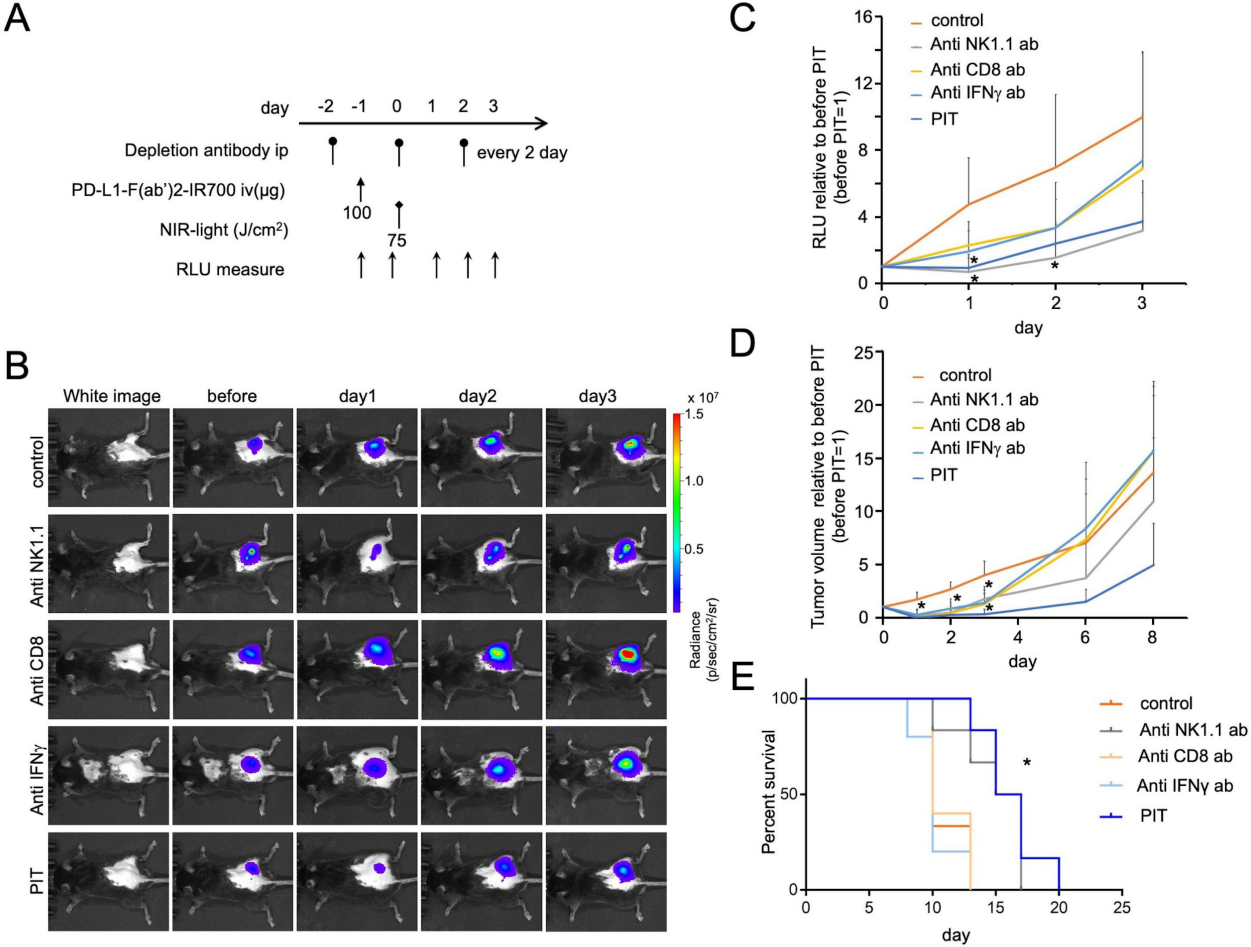
PD-L1-targeted NIR-PIT induces antitumor effects via partially on CD8 T and NK cells and IFN-γ production. (A) PD-L1-targeted NIR-PIT regimen involving PD-L1-F(ab′)_2_-IR700 injection, intraperitoneal injection of neutralized antibodies, and NIR-light exposure is shown. Depletion antibodies were injected every 2 days. (B) Representative in vivo BLI of tumor-bearing mice (right flank tumor). (C) Quantitative RLU showed a significant decrease in PD-L1-targeted NIR-PIT-treated tumors but was inhibited by adding the depletion or neutralization antibodies (n=5–6 in each group) (control group vs PIT and anti NK1.1 group at day 1: *p<0.01; control group vs anti NK1.1 group at day 2: *p<0.05, Tukey’s test with ANOVA). (D) Tumor volume ratio (before NIR-PIT=1) is demonstrated. NIR-PIT introduced on day 0 led to significant reductions in the tumor volume ratio but inhibited with adding the depletion or neutralization antibodies (n=5–6 in each group) (control group vs all other groups at day 1: *p<0.0001; control group vs anti CD8 and anti NK1.1 groups at day 2: *p<0.001; control group vs anti IFN-γ group at day 2: *p<0.01; control group vs PIT group at day 2: *p<0.0001; control group vs anti-CD8 group at day 3: *p<0.05; control group vs anti-NK1.1 and anti IFN-γ groups at day 3: *p<0.01; control group vs PIT group at day 3: *p<0.001, Tukey’s test with ANOVA). (E) The survival of PD-L1-targeted NIR-PIT was shortened by adding the depletion or neutralization antibodies (n=5–6 in each group) (*p=<0.001 (0.0007), log-rank test). ANOVA, analysis of variance; BLI, bioluminescence imaging; IFN-γ, interferon-gamma; NK, natural killer; NIR-PIT, near-infrared photoimmunotherapy; PD-L1, protein programmed death-ligand 1; RLU, relative light unit.

To elucidate the role of effector cells in PD-L1-targeted NIR-PIT, we depleted NK or CD8 T cells with repeated systemic injections of anti-NK1.1 or anti-CD8 antibody, respectively, or neutralized IFN-γ by repeatedly administering anti-IFN-γ antibody (figure 5A). NK or CD8 T cell depletion, or IFN-γ neutralization attenuated the PD-L1-targeted NIR-PIT efficacy, as demonstrated by the quantified luciferase activity, and tumor growth and mouse survival data (figure 5B–E). These data demonstrated that antitumor efficacy of PD-L1-targeted NIR-PIT was mediated, at least partly, by NK cells, CD8 T cells, and IFN-γ production; it was also likely to be mediated by a combination of all these factors.

## Discussion

Strategies involving ICIs, such as PD-L1 antibody-based therapy, are evidently effective in treating various cancers; thus, it is now widely used clinically.^5^ However, the response rate is limited (approximately 10%–30%), with a need for improvement. Therefore, we should evaluate combining ICIs with other modalities, possibly making anticancer immunotherapy more effective.

**Figure 6:**
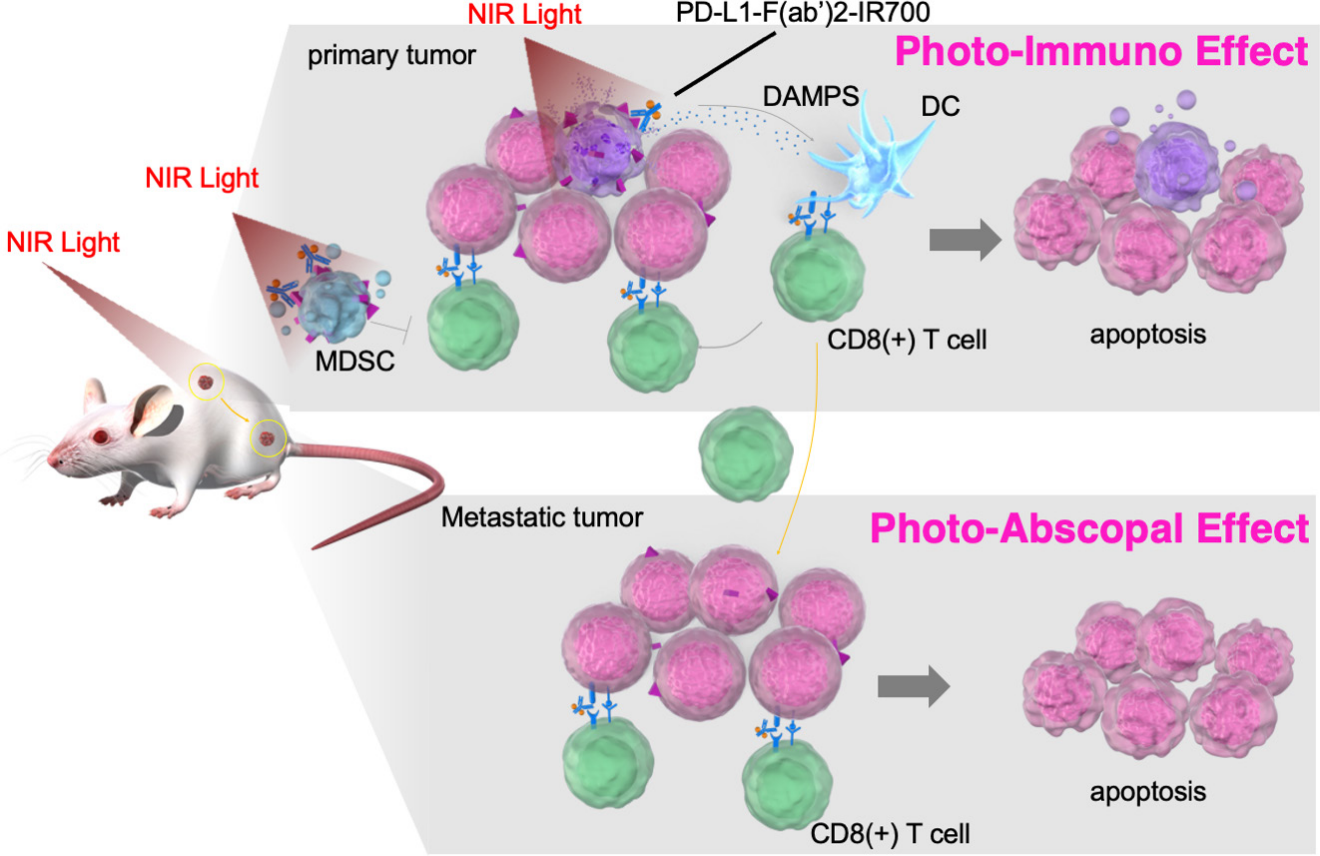
Scheme: the proposed mechanism of local PD-L1-targeted NIR-PIT–induced cancer immunotherapy. Near-infrared photoimmunotherapy targeting of PD-L1 showed antitumor effects via the following pathways: (1) direct photocytotoxicity; (2) releasing damage-associated molecular patterns; (3) depleting MDSCs, (4) activating CD8 T cells and NK cells; and (5) PD-L1-F(ab′)_2_-IR700 blocks the PD-1/PD-L1 axis. This antitumor immune enhancement leads to unexpected regression of the treated tumor, whereas PD-L1 expression on the tumor was low. These antitumor immune augmentations systematically induce photo-abscopal effects on distant tumors. MDSCs, myeloid-derived suppressor cells; NIR-PIT, near-infrared photoimmunotherapy; PD-1, programmed death-1; PD-L1, programmed death-ligand 1.

Although NIR-PIT is considered a promising treatment, it has some limitations, including its need for high expression of cancer target cell-surface antigens, the inability to overcome treatment resistance due to the heterogeneous intratumoral antigen expression, and it being only a localized treatment and not having an expanded effect on metastatic areas. To overcome these shortcomings, we exploited PD-L1 as a target for the tumor cell itself and tumor microenvironment editing. PD-L1 is usually not highly expressed on tumor cells; however, it is almost expressed at low levels in any tumor cell across all organs.^6^ Moreover, blocking the PD-1 signaling axis by targeting PD-L1 also enables the ICI effect. PD-L1-targeted NIR-PIT showed an unexpectedly strong therapeutic effect in vivo, despite its limited photocytotoxicity in vitro. In the treated tumors, activated CD8^+^ T and NK cells were observed immediately after treatment, suggesting that in addition to directly damaging the tumor cells, NIR-PIT activated antitumor immunity. Moreover, PD-L1-targeted NIR-PIT reduced the intratumoral MDSC number, without affecting effector cell counts. It has been reported that aggregation of IR700 conjugates leads to cell membrane disruption and influx of the surrounding aqueous solution, resulting in cell necrosis.^37^ Consequently, unlike conventional cancer therapies, which generally induce apoptotic cell death, the IR700 conjugate-based strategy induces selective immediate immunogenic cell death, and the release of tumor antigens and DAMPs from the necrotic tumor leads to activating APCs, such as dendritic cells.^38^ Here, NIR-PIT also resulted in the release of tumor cell DAMPs. These antitumor effects were partially reduced by depleting CD8 T or NK cells or neutralizing IFN-γ. In the in vivo model of bilateral tumor implantation, activated CD8 T and NK cells were observed in the tumor on the NIR-PIT-treated side and the contralateral side (nonirradiated tumor), leading to antitumor effects on untreated tumors (photo-abscopal effect). The release of DAMPs and other substances following tumor necrosis, combined with alteration in the tumor microenvironment, and the immune checkpoint inhibition by the anti-PD-L1 antibody may enhance tumor immune response. Therefore, this strategy could be effective against metastatic tumor cells (figure 6).

ICIs are nowadays favorably combined with radiotherapy (RT) or cytotoxic chemotherapy, rather than being applied as monotherapy. For example, durvalumab is approved for use after chemoradiotherapy for unresectable progressive non-small cell lung carcinoma and in combination with cytotoxic chemotherapy for small-cell lung carcinoma.^39^ RT causes tumor cell death and releases tumor debris, danger signals, tumor-associated antigens, and inflammatory cytokines, stimulating innate immune cells such as dendritic cells. After presenting the tumor antigen in the lymph nodes, antigen-specific T cells emerge and attack the original tumor or metastatic tumors. This immune activation may be enhanced by systemic immunotherapy.^40^ Anti-PD-L1 antibodies alone are poorly cytotoxic; however, when combined with RT, they upregulate tumor cell MHC and FAS expression and enhance sensitivity to T cell cytotoxicity.^33^ Furthermore, RT enhances PD-L1 expression in tumors with inflammation,^41^ and distant tumors shrink following combination treatment with RT and PD-L1 antibody.^42^ Similarly, NIR-PIT can also enhance antitumor effects with ICIs. RT targets tumor cells and damages all irradiated immune cells. However, as NIR-PIT can induce highly selective tumor cell cytotoxicity with minimal damage to other cells, antitumor immune reactions could be more enhanced with NIR-PIT than with RT.

Recent studies revealed that the tumor microenvironment majorly affects tumor growth.^43^ Anticancer therapies targeting or altering the tumor microenvironment may be a promising new treatment stream.^44^ Targeting tumor microenvironments with NIR-PIT has been performed with some success, such as with intratumoral Tregs, cancer-associated fibroblasts, and intratumoral vessels.^21 45 46^ MDSCs are a heterogeneous collection of myeloid precursor cells, and immature granulocytes, macrophages, and dendritic cells.^47^ In cancer, MDSCs play a role in immune surveillance evasion by interacting with tumor and other stromal cells.^48^ Increased MDSC counts are reportedly associated with worsened tumor progression, increased severity, and poor prognosis in lung cancer patients.^49^ Here, intratumoral MDSC counts were reduced following PD-L1-targeted NIR-PIT, and this reduction might have enhanced local tumor immunity. Therefore, PD-L1-targeted NIR-PIT could alter the tumor bed, augmenting the antitumor immune response.

NIR-PIT targeting PD-L1, has several advantages. First, treatment with anti-PD-L1 antibodies is effective for tumors in many different organs.^5^ Thus, PD-L1-targeted NIR-PIT can be used regardless of tumor type, making this technology suitable for more number of patients. Second, treatment with anti-PD-L1 antibodies is effective even with low tumor PD-L1 expression, as shown herein. Third, anti-PD-L1-targeted NIR-PIT is expected to have therapeutic effects on NIR light irradiated-tumors and distant tumors not directly irradiated with NIR light—the so-called photo-abscopal effect. Contrastingly, conventional NIR-PIT can only be applied to locally treat advanced tumors, which is its biggest flaw. Fourth, a number of anti-PD-L1 antibody drugs have already been approved by the US Food and Drug Administration and are applied clinically. Therefore, PD-L1-targeted NIR-PIT could easily be clinically translated. Finally, PD-L1-targeted NIR-PIT was able to activate PD-L1 on the treated tumor cells, making PD-L1-targeted therapy more effective on repetition. With inflammation due to photocytotoxicity of tumor cells, cytokines such as INF-γ promote PD-L1 expression.^34^

There are a few potential concerns regarding this study. APCs, such as dendritic cells and macrophages, are key players in the anticancer immune system. These APCs also express PD-L1; thus, PD-L1-targeted NIR-PIT may reduce the number of local APCs that mainly work in the lymph nodes, presenting tumor antigens to T cells. However, NIR light does not irradiate these lymph nodes; therefore, the NIR-PIT effect on APCs is considered negligible. Moreover, immune cells can move to non-irradiated lesions in the body. Next, in vivo experiments indicated that the therapeutic effect might be temporary. This therapy can be repeated,^50^ and ICIs can be used on NIR-PIT inflammation-induced PD-L1 expression; therefore, repeated therapy or combination therapy with ICIs could be used for recurrent tumors. Third, the depth to which NIR can penetrate with clinically meaningful intensity is limited^51^ so tumors located deep inside the body cannot be treated from the surface of the body with NIR-PIT. However, in addition to extracorporeal irradiation, transcatheter NIR irradiation devices^52 53^ and implantable devices have also been developed.^54^ It has the potential to treat tumors on the surface of the body and a variety of cancers deep inside body with endoscopes and other techniques. Finally, injury to the PD-L1-expressing tissues surrounding tumor may occur. However, we can minimize the injury to the normal tissues, because in clinical, the treatment area can be determined in advance by CT or other means (MRI, ultrasound, etc). Since areas without exposure to NIR-light are not damaged, and since we can target and limit the area to be irradiated, the injury to the non-tumor site can be minimized. Due to its high selectivity, the damage to normal tissues by NIR-PIT is likely to be much narrower than that by systemic chemotherapy, surgery, or RT.

In conclusion, we demonstrated that PD-L1-targeted NIR-PIT directly damaged tumors locally and activated CD8 T and NK cells, resulting in local and systemic tumor therapeutic effects. NIR-PIT targeting PD-L1 could be an effective systemic treatment for various tumors.

10.1136/jitc-2021-003036.supp2 Supplementary data

## Data Availability

Data are available on reasonable request. Data and materials are available upon reasonable request. All data relevant to the study are included in the article or uploaded as supplementary information.
