# Supplementary material for: Spatiotemporal depletion of tumor-associated immune checkpoint PD-L1 with near-infrared photoimmunotherapy promotes antitumor immunity
Source: J Immunother Cancer. 2021 Oct 28;9(11):e003036. doi: 10.1136/jitc-2021-003036 (PMC8559243; doi:10.1136/jitc-2021-003036)
Supplement: Supplementary data [file jitc-2021-003036supp001.pdf]

Supplementary Information

Spatiotemporal depletion of tumor-associated immune checkpoint PD-L1 with near infrared photoimmunotherapy promote antitumor immunity

Shunichi Taki[1,#], Kohei Matsuoka[2], Yuko Nishinaga[1], Kazuomi Takahashi[1], Hirotoshi Yasui[1], Chiaki Koike[3], Misae Shimizu[3], Mitsuo Sato[2], Kazuhide Sato[1,3,4,5,6 # *]

[1] Respiratory Medicine, Nagoya University Graduate School of Medicine
[2] Department of Integrated Health Sciences, Nagoya University Graduate School of Medicine, Nagoya, Japan
[3] Nagoya University Institute for Advanced Research, Advanced Analytical and Diagnostic Imaging Center (AADIC) / Medical Engineering Unit (MEU), B3 Unit
[4] Nagoya University Institute of Nano-Life-Systems, Institutes of Innovation for Future Society
[5] FOREST-Souhatsu, CREST, JST
[6] Nagoya University Institute for Advanced Research, S-YLC
[#] These authors contributed equally

＊Correspondence should be addressed to: Kazuhide Sato, M.D., Ph.D. Institute for Advanced Research, Department of Respiratory Medicine, Graduate School of Medicine, Nagoya University, Nagoya, Aichi, 466-8550, Japan,
Phone: +81-052-744-2167; Fax: +81-052-744-2176;
E-mail: k-sato@med.nagoya-u.ac.jp

Supplementary Figure 1

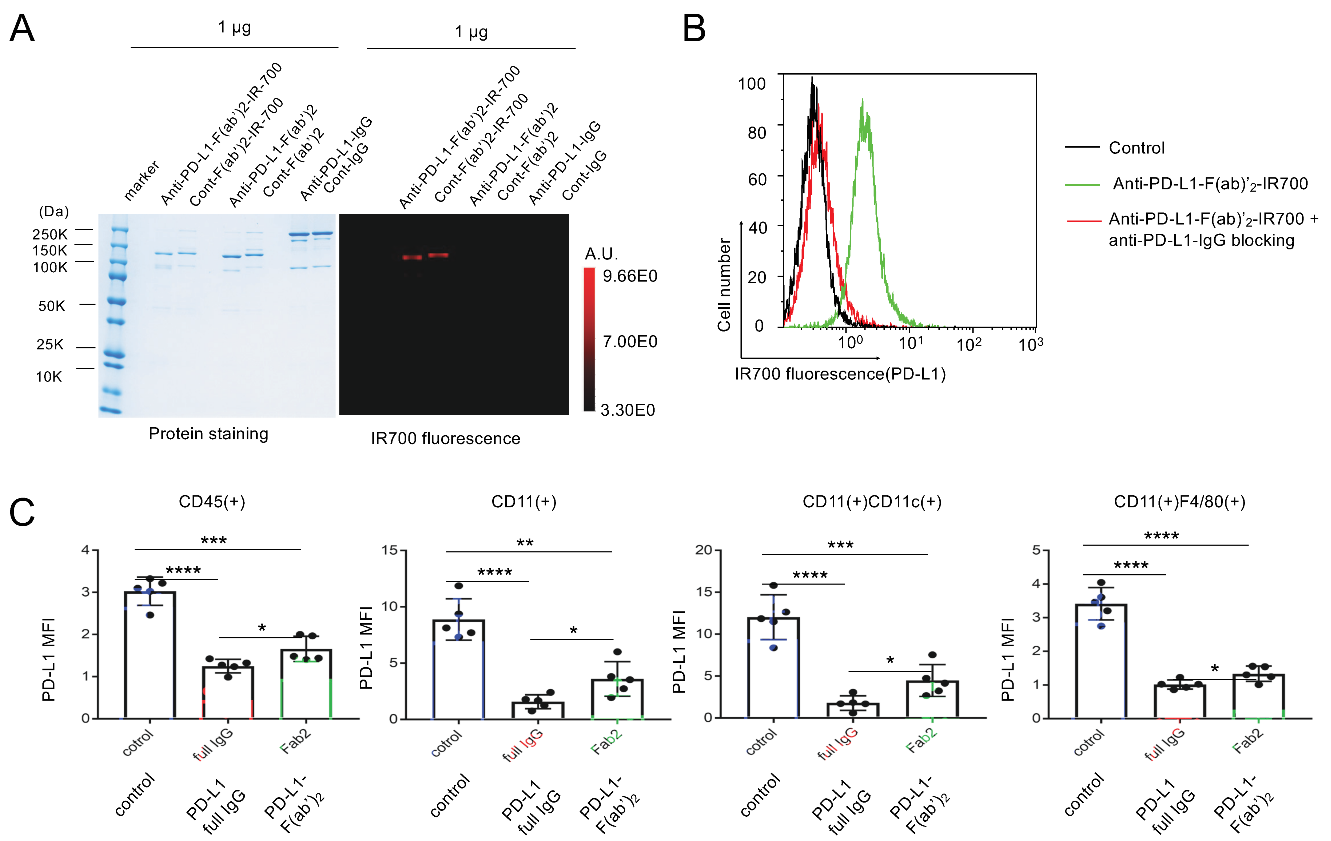

Supplementary Figure 1
Production of Fc-deficient anti-PD-L1- F(ab')$_2$–IR700 and evaluation *in vivo.*

(A) Validation of conjugated PD-L1-F(ab')$_2$-IR700 with SDS-PAGE (left: colloidal blue staining, right: 700 nm-fluorescence image).
(B) PD-L1-F(ab')$_2$-IR700 specifically bind on PD-L1. To block the PD-L1 on the PD-L1-overexpressed LL/2, excess anti-PD-L1-IgG was added on the medium. After incubation with anti-PD-L1-IgG, PD-L1-F(ab')$_2$-IR700 was then added.
(C) Evaluation of the effect in the spleen of intravenous administration of either anti-PD-L1-IgG or anti-PD-L1-F(ab')$_2$. Anti PD-L1 full IgG suppressed PD-L1-expression on antigen-presenting cells (APCs) in the spleen more strongly than anti PD-L1 F(ab')2. Data are means ± SEMs (n = 5, $^{*}p < 0.05$, $^{**}p < 0.01$, $^{***}p < 0.001$, $^{****}p < 0.0001$, Student's t-test)

Supplementary Figure 2

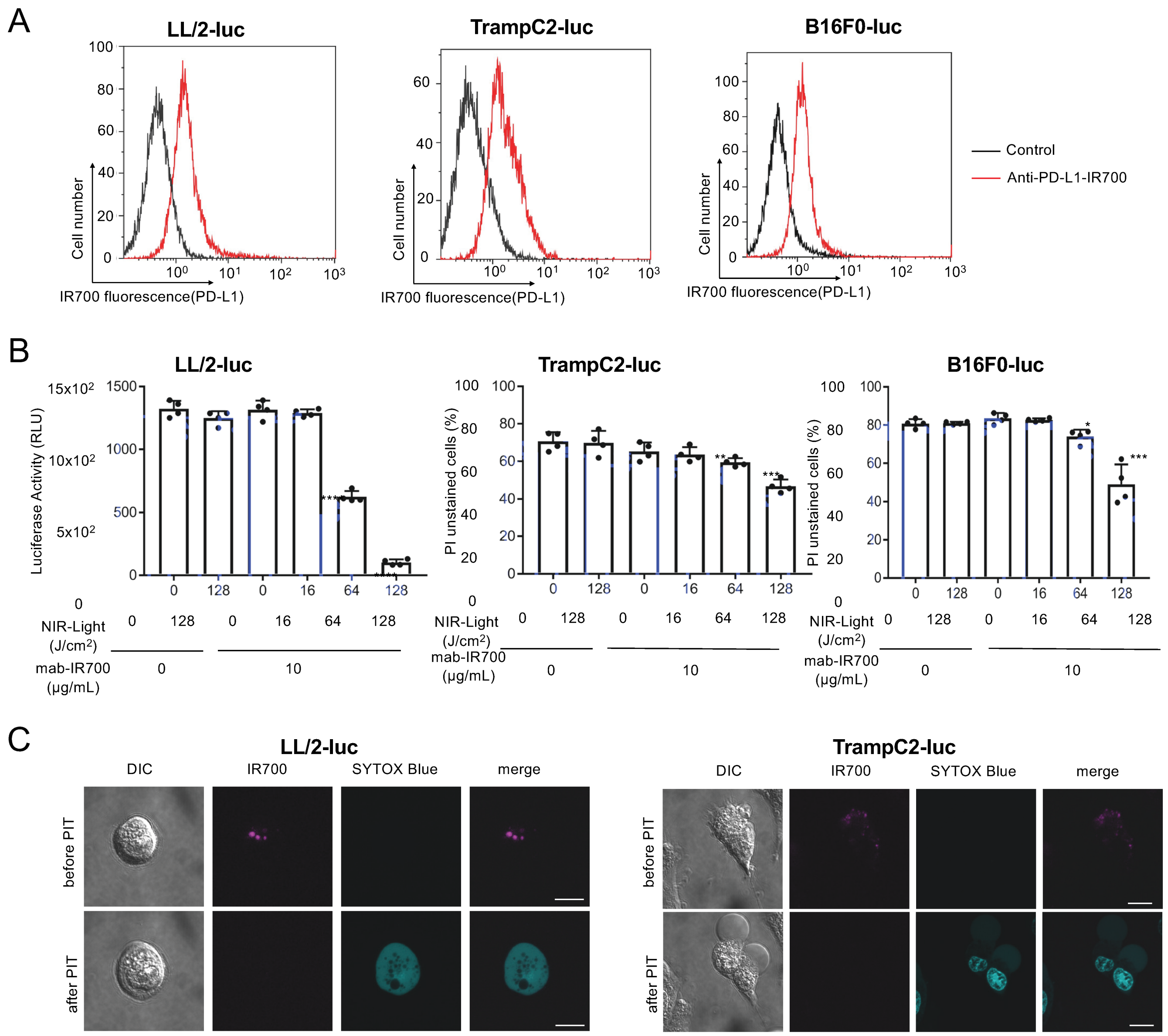

Supplementary Figure 2

***In vitro* assessment of PD-L1-expression, and evaluation of *in vitro* NIR-PIT with anti-PD-L1- F(ab')$_2$–IR700 on various murine tumor cells.**

(A) PD-L1-expression in various murine cancer cell lines (LL/2 (lung cancer), Tramp-C2 (prostate cancer), B16F$_0$ (melanoma)) by flow cytometery. Low expression of PD-L1 was universally detected on the cell lines.

(B) *In vitro* cytotoxicity after PD-L1-targeted NIR-PIT was measured by counting dead cells stained with propidium iodide or luciferase activities, which increased in a NIR-light-dose dependent manner. Data are means ± SEMs (n = 4, * p < 0.05, ** p < 0.01, ***p < 0.001, ****p < 0.0001, Student's t-test)

(C) Microscopic observations before and after *in vitro* PD-L1-targeted NIR-PIT. LL/2-luc or MC38-luc cells were incubated with PD-L1-F(ab')2-IR700 for 6 hours and observed with a microscope before and after irradiation with NIR-light (4 J/cm^2). Necrotic cell death which was stained with SYTOX Blue Dead Cell Stain, was observed after exposure on NIR-light at 20 minutes after NIR-PIT (scale bar, 10 μm).

Supplementary Figure 3

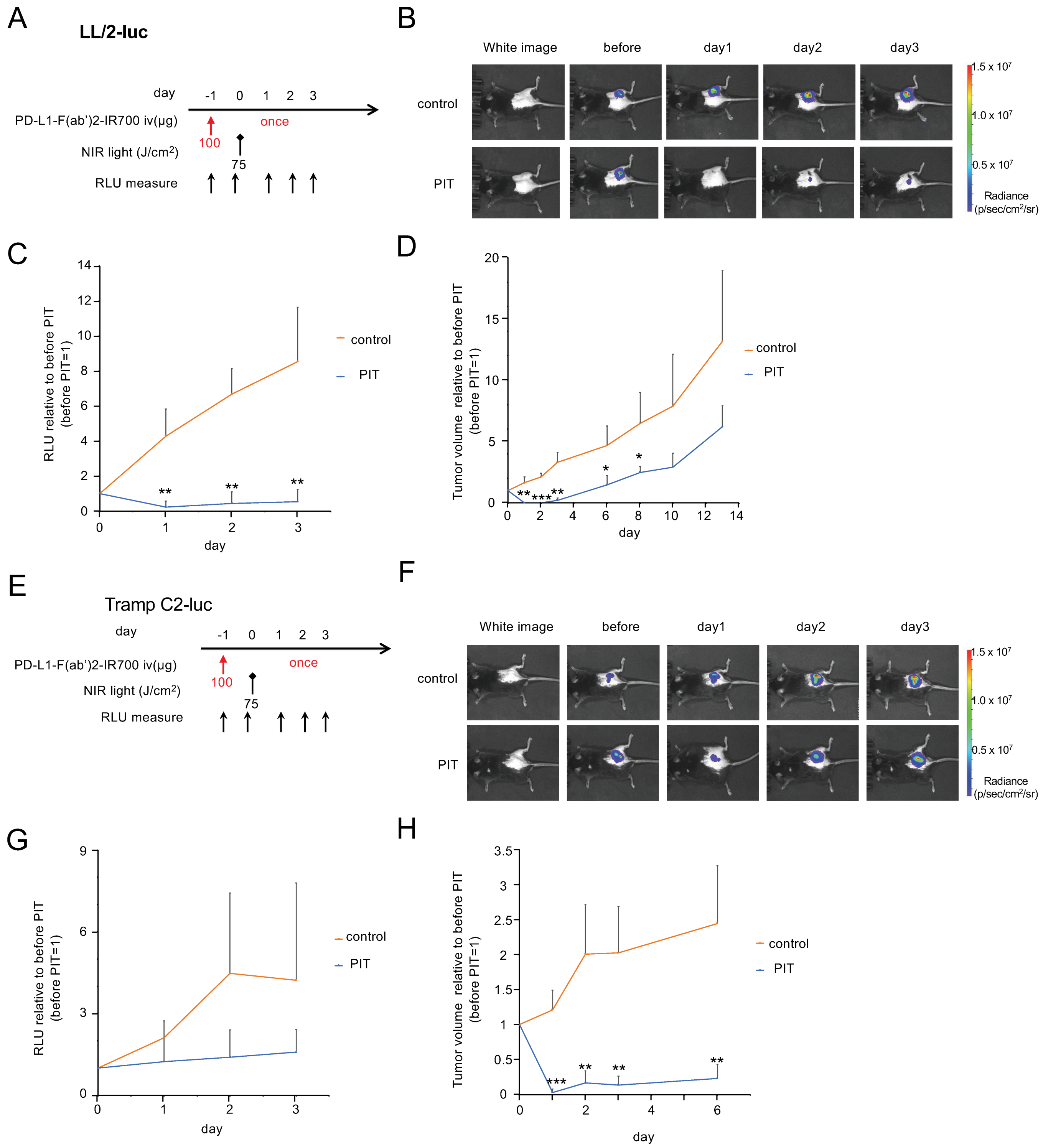

Supplementary Figure 3

***In vivo* NIR-PIT targeting intratumoral PD-L1 induces unexpected remarkable regression of treated various murine tumors.**

(A) PD-L1-targeted NIR-PIT regimen involving PD-L1-F(ab')2-IR700 injection, BLI acquisition and NIR-light exposure is indicated.
(B) *In vivo* BLI of LL/2-luc tumor-bearing mice. In the NIR-PIT group, luciferase activity was decreased after irradiation with NIR-light.
(C) Quantitative RLU showed a significant decrease in PD-L1-targeted NIR-PIT-treated LL/2-luc tumors. Data are means ± SD (n = 3-4, $^{**}p < 0.01$, Student's t-test)
(D) Tumor volume ratio (defined as before NIR-PIT = 1) demonstrated that NIR-PIT introduced on day 0 led to significant reductions in the LL/2-luc tumor volume. Data are means ± SD (n = 4, $^{*}p < 0.05$, $^{**}p < 0.001$, $^{***}p < 0.0001$, Student's t-test)
(E) *In vivo* BLI of Tramp C2-luc tumor-bearing mice. In the NIR-PIT group, luminescence was decreased after irradiation with NIR-light.
(F) Quantitative RLU showed a decrease in PD-L1-targeted NIR-PIT-treated Tramp C2-luc tumors. Data are means ± SD (n = 3-4)
(G) Tumor volume ratio (defined as before NIR-PIT = 1) demonstrated that NIR-PIT introduced on day 0 led to significant reductions in the Tramp C2-luc tumor volume. Data are means ±SD (n = 3-4, $^{**}p < 0.001$, $^{***}p < 0.0001$, Student's t-test).

Supplementary Figure 4

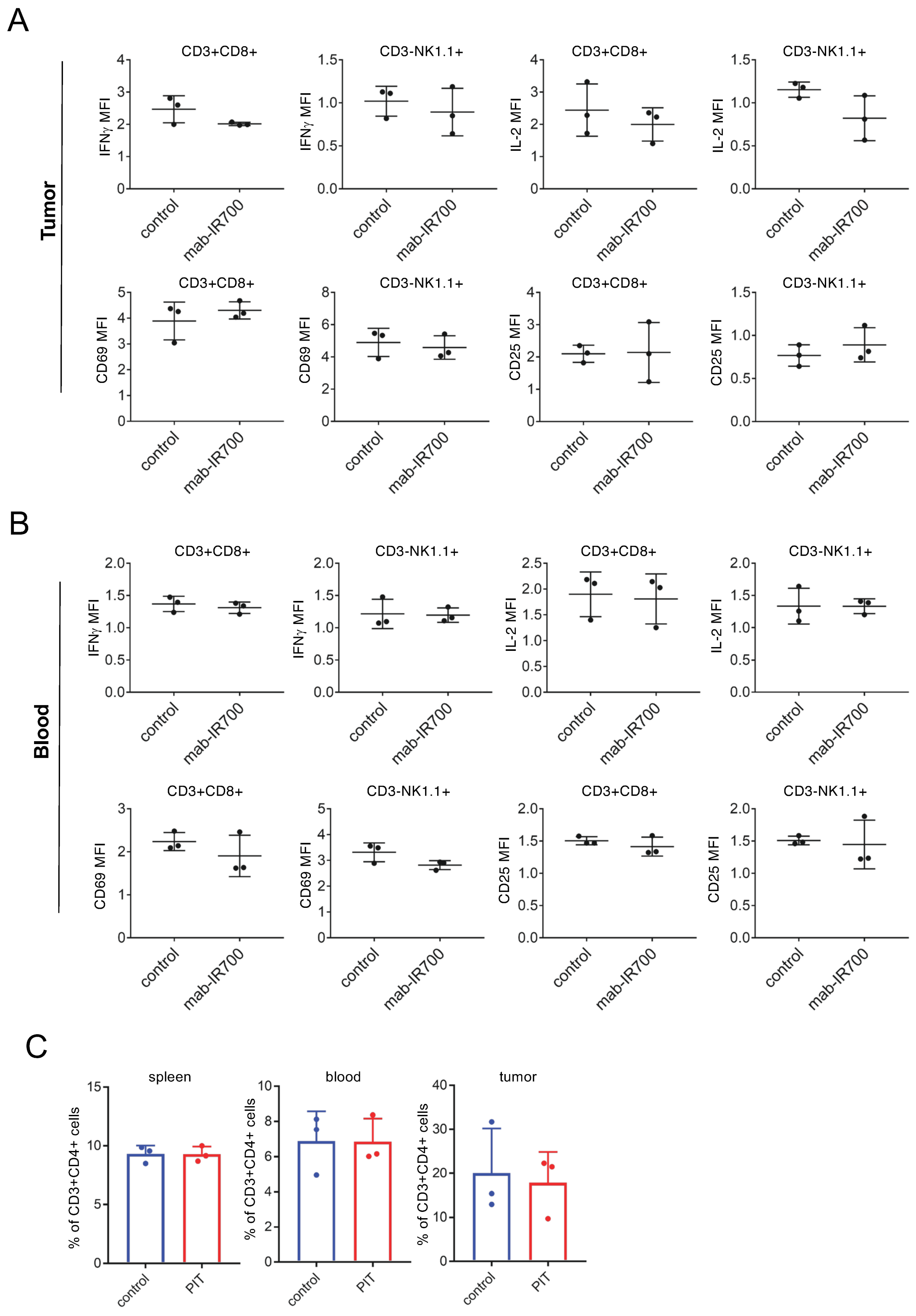

Supplementary Figure 4

In vivo **injection of PD-L1-F(ab')2-IR700 induce no activation of tumor-infiltrating CD8 T and NK cells, and PD-L1-targeted NIR-PIT did not affect on $CD4^+CD25^+Foxp3^+$ regulatory T cells (Tregs).**

(A)(B) Cytotoxic action of intra-tumoral (A) and peripheral blood (B) CD8 T and NK cells was examined by flow cytometry with or without PD-L1-F(ab')2-IR700 injection. (n = 3; no significant difference in each activation marker, Student's t-test)
(C) Quantitative data of the percentage of $CD25^+FOXP3^+$Treg in $CD4^+$ cells in spleen, blood, or tumor, at immediately after PD-L1-targeted NIR-PIT was showed (n = 3; no significant difference, Student's t-test).

Supplementary Figure 5

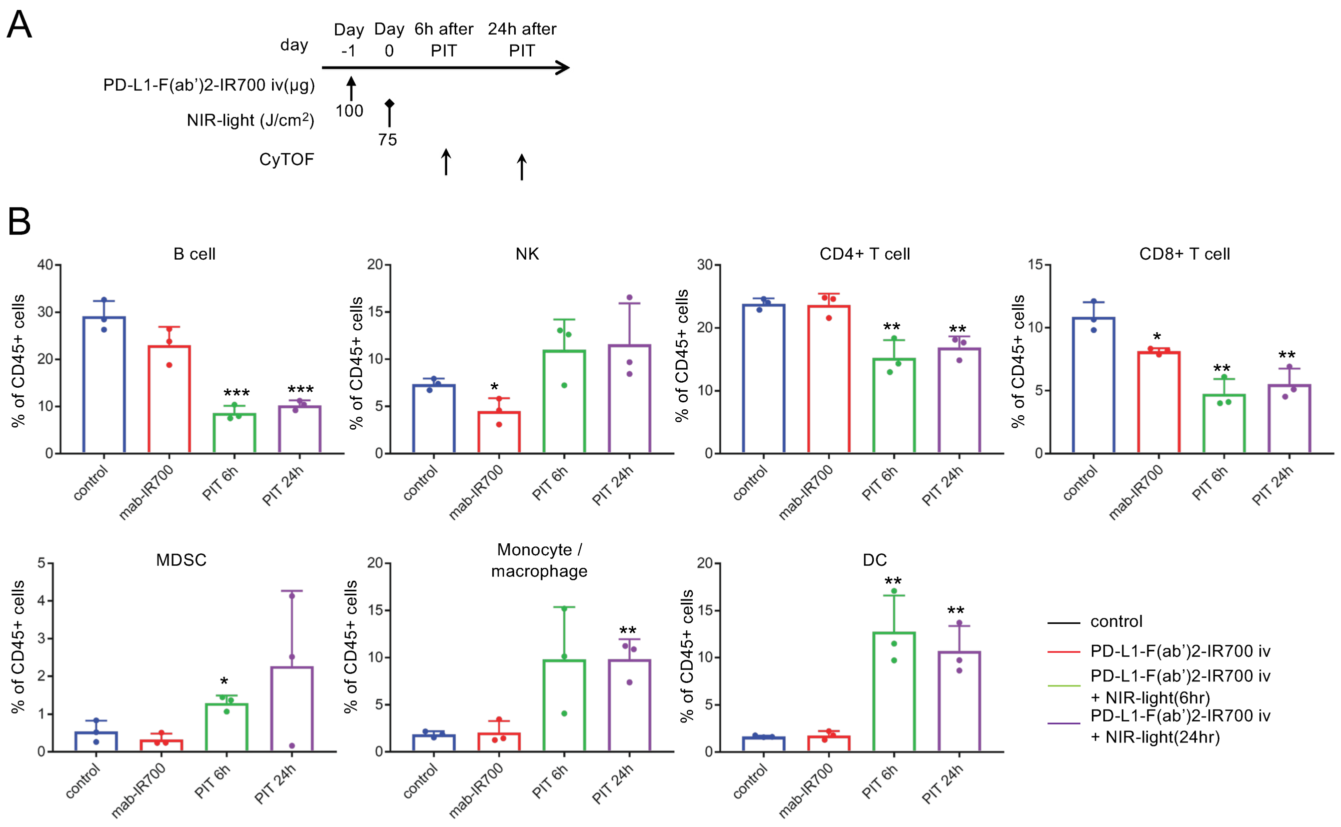

Supplementary Figure 5

(A) PD-L1-targeted NIR-PIT regimen for CyTOF analysis in blood is indicated. To acquire the immune biomarker, peripheral blood in mice at 6 or 24 hours after the PD-L1-targeted NIR-PIT was analyzed.

(B)Quantitative data of the percentage of CD45+ cells in blood by CyTOF analysis. (n = 3; *p<0.05, **p<0.01, Student's t-test).

Supplementary Figure6

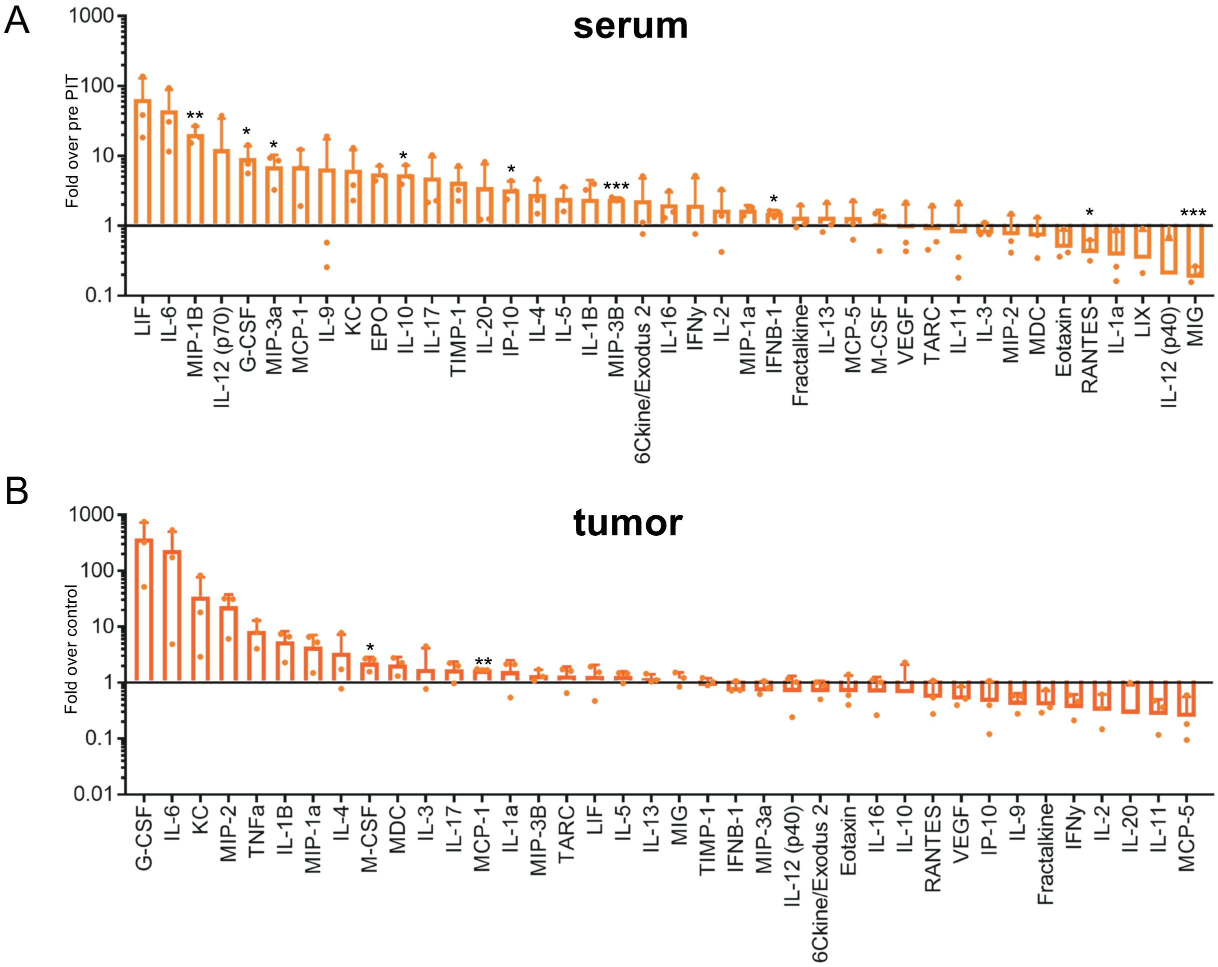

Supplementary Figure 6

PD-L1-targeted NIR-PIT induces acute production of cytokines and chemokines in serum and treated tumor.

(A) Cytokine and chemokine in the serum before and at 6 hours after NIR-light irradiation in the PD-L1-targeted NIR-PIT treated tumor were sequentially measured in each mouse. The results are indicated as fold increase (n = 3, means ± SEMs $^{*}p < 0.05$, $^{**}p < 0.01$, $^{***}p < 0.001$, Student's t-test).

(B) Cytokine and chemokine in the tumor were accessed at 24 hours after NIR-light irradiation and then, compared to that in untreated tumors. The results are indicated as fold increase (n = 3, means ± SEMs $^{*}p < 0.05$, $^{**}p < 0.01$, Student's t-test).

Supplementary Figure7

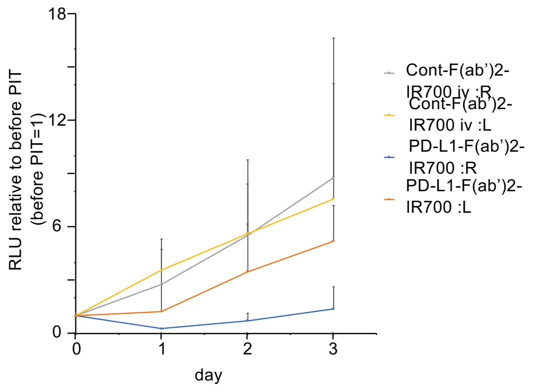

Supplementary Figure 7

The therapeutic effects of PD-L1-targeted NIR-PIT could extend to distant non-NIR-light irradiated tumor; inducing "abscopal-effects".
Quantitative RLU showed decrease in both non-irradiated left tumors and NIR-light irradiated tumors with PD-L1-F(ab')2-IR700 administration, however, the decrease was not significant. (n = 3 in each group , means ± SD, no significant difference, Tukey's test with ANOVA)

Supplementary Figure8

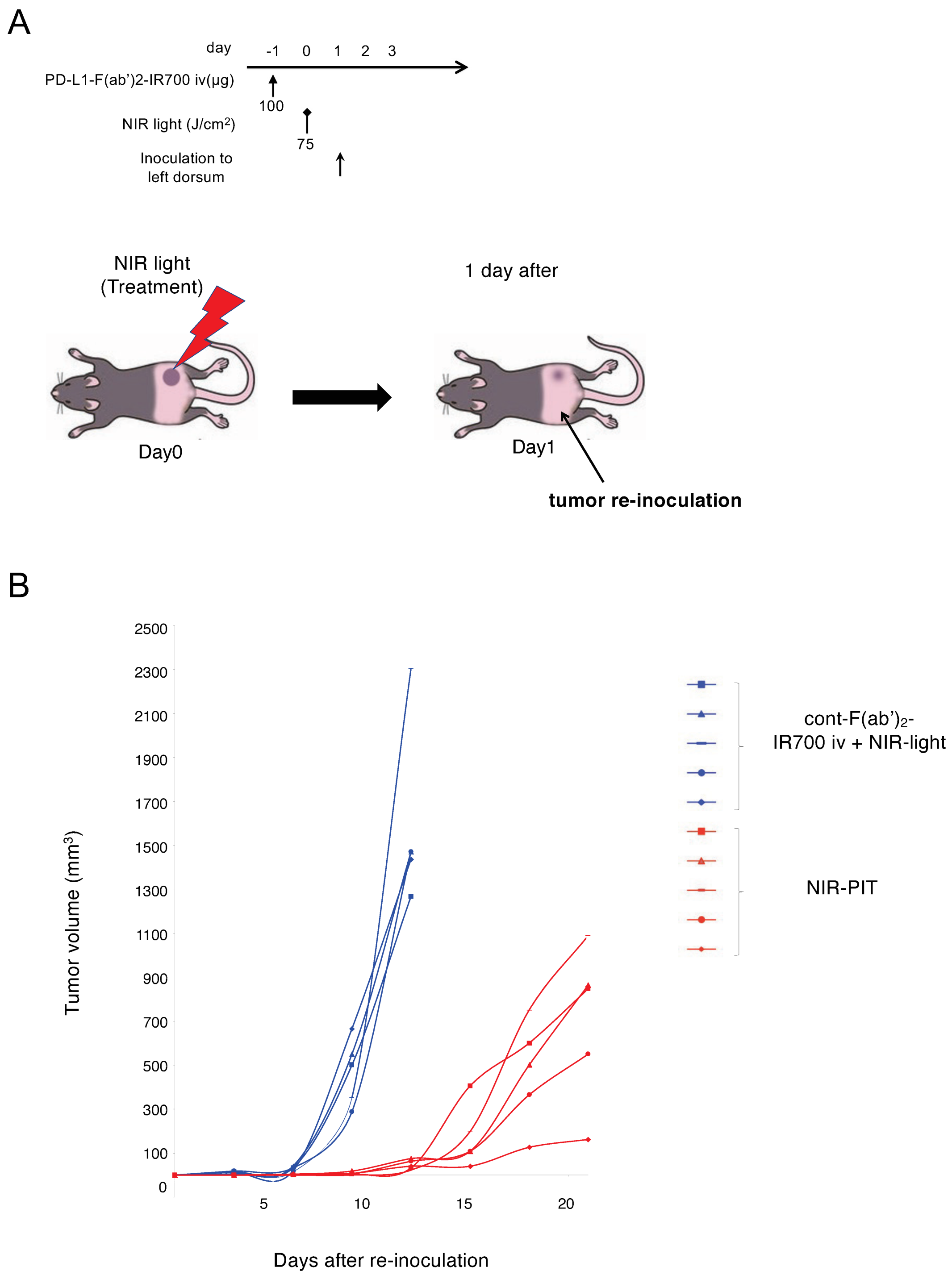

Supplementary Figure 8

Local PD-L1-targeted NIR-PIT inhibited the growth of the tumor challenged on the contralateral side.

(A) The procedure regimen is shown. A schematic representation is also demonstrated.

(B) The growth of MC38-luc tumor inoculated on the contralateral side 1 day after local PD-L1-targeted NIR-PIT of the same tumor type was inhibited compared to that inoculated into control mice received with control-F(ab')2-IR700 administration with NIR light irradiation (n =5 per in each group).
