# Supplementary figures and images for: Spatiotemporal depletion of tumor-associated immune checkpoint PD-L1 with near-infrared photoimmunotherapy promotes antitumor immunity
Source: J Immunother Cancer. 2021 Oct 28;9(11):e003036. doi: 10.1136/jitc-2021-003036 (PMC8559243; doi:10.1136/jitc-2021-003036)

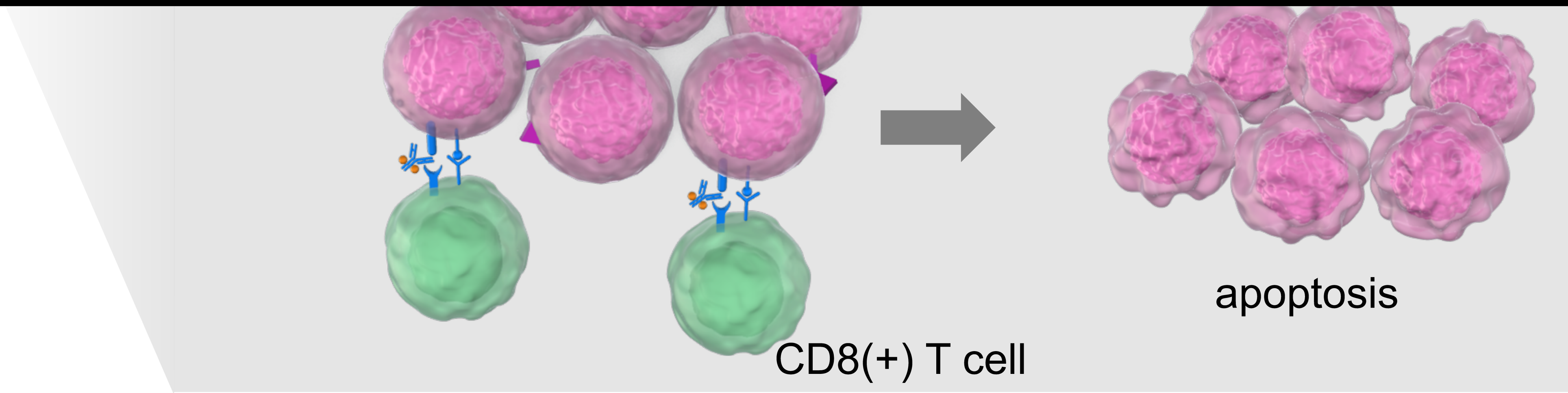

Supplement: Supplementary data [file jitc-2021-003036supp002.pdf]
